# Olfactory Projections to Locomotor Control Centers in the Sea Lamprey

**DOI:** 10.3390/ijms25179370

**Published:** 2024-08-29

**Authors:** Philippe-Antoine Beauséjour, Jean-Christophe Veilleux, Steven Condamine, Barbara S. Zielinski, Réjean Dubuc

**Affiliations:** 1 Department of Neurosciences, Faculty of Medicine, University of Montreal, Montreal, QC H3T 1J4, Canada; philippe-antoine.beausejour@umontreal.ca (P.-A.B.); s.condamine@gmail.com (S.C.); 2 Research Group in Adapted Physical Activity, Department of Exercise Sciences, Faculty of Sciences, University of Quebec in Montreal, Montreal, QC H2X 1Y4, Canada; christophe.roy@fizl.io; 3 Department of Integrative Biology, Faculty of Science, University of Windsor, Windsor, ON N9B 3P4, Canada; zielin1@uwindsor.ca

**Keywords:** olfaction, locomotion, dopamine, motor system, neuroanatomy, electrophysiology, calcium imaging, lamprey

## Abstract

Although olfaction is well known to guide animal behavior, the neural circuits underlying the motor responses elicited by olfactory inputs are poorly understood. In the sea lamprey, anatomical evidence shows that olfactory inputs project to the posterior tuberculum (PT), a structure containing dopaminergic (DA) neurons homologous to the mammalian ventral tegmental area and the substantia nigra pars compacta. Olfactory inputs travel directly from the medial olfactory bulb (medOB) or indirectly through the main olfactory bulb and the lateral pallium (LPal). Here, we characterized the transmission of olfactory inputs to the PT in the sea lamprey, *Petromyzon marinus*. Abundant projections from the medOB were observed close to DA neurons of the PT. Moreover, electrophysiological experiments revealed that PT neurons are activated by both the medOB and LPal, and calcium imaging indicated that the olfactory signal is then relayed to the mesencephalic locomotor region to initiate locomotion. In semi-intact preparations, stimulation of the medOB and LPal induced locomotion that was tightly associated with neural activity in the PT. Moreover, PT neurons were active throughout spontaneously occurring locomotor bouts. Altogether, our observations suggest that the medOB and LPal convey olfactory inputs to DA neurons of the PT, which in turn activate the brainstem motor command system to elicit locomotion.

## 1. Introduction

Mesodiencephalic dopaminergic (DA) neurons play an important role in motor control through modulation of the basal ganglia. In the brain of the lamprey, which occupies an important phylogenetic position as the oldest extant group of vertebrates, i.e., the Agnatha, the substantia nigra pars compacta and ventral tegmental area (SNc/VTA) homolog is called the nucleus of the posterior tuberculum (PT) and contains a high density of DA neurons [1,2,3,4,5,6,7,8,9]. The considerable similitude of the lamprey PT to the SNc/VTA, including connectivity, cellular properties, and behavioral effects of DA, suggests that this DA system appeared before the separation of the lamprey and mammalian lineages; reviewed in [10]. Notably, ascending DA projections to the direct and indirect basal ganglia pathways exist in lampreys [7,8,11,12]. Parkinson’s disease has been traditionally associated with the loss of these DA projections [13,14,15,16,17]; for reviews, see [18,19,20]. Interestingly, using the lamprey model, our research group has shown that DA neurons in the PT do not only project rostrally but they also send descending projections that reach a brainstem region playing a crucial role in controlling locomotion, the mesencephalic locomotor region (MLR) [21,22]. In addition to the descending DA projections to the MLR, DA projections that directly modulate motor output were also found to reach brainstem motor nuclei, such as the optic tectum [8,9] as well as reticulospinal (RS) cells in the hindbrain [23]. Importantly, we have shown that electrical and pharmacological stimulation of the PT induces swimming in a semi-intact lamprey preparation with the tail freely moving in a recording chamber [21,22,23,24], suggesting a crucial function of this brain region in locomotor control. Furthermore, the PT receives axonal projections from many sensory regions [7] and responds to visual and electrosensory inputs [8]. Therefore, as in mammals, the activity of DA neurons in the PT (or SNc/VTA) may be influenced by multiple forms of sensory inputs and, in turn, exert a direct DA modulation on motor nuclei.

Because the PT receives projections from the olfactory bulb [7,24,25,26,27,28,29,30,31,32], the PT is also likely to be activated by olfactory inputs, which are crucial to guide behaviors, such as feeding and reproduction, in lampreys. For instance, odor detection enables adult lampreys to locate prey [33] as well as spawning grounds [34]. Moreover, the application of specific odor cues on the olfactory epithelium or electrical stimulation of the olfactory nerve and bulb activates RS cells [24,35,36], which in turn provide excitatory input to the spinal locomotor networks [37,38,39,40,41,42]. Anatomical and physiological experiments from our research group laid the groundwork for the identification of neural pathways linking odor detection to behavior in the sea lamprey, *Petromyzon marinus*; reviewed in [43]. First, in a medial pathway, peripheral input from the accessory olfactory organ is transmitted to the medial OB (medOB), which has direct projections to the PT [24]. Second, in a lateral pathway, the main olfactory epithelium conveys information to the main OB (MOB) that then reaches the PT via projections to the lateral pallium (LPal) [30]. Stimulation of the LPal was previously shown to induce swimming in the lamprey [44]. It is presumed that in both pathways, descending projections from the PT activate the MLR, which exerts powerful control over RS cell activity ([45,46], reviewed in [47,48,49,50]), and thus, locomotion. Indeed, since pharmacological inactivation of the PT decreases subthreshold excitatory postsynaptic potentials in RS cells induced by olfactory nerve [24] or LPal [30] stimulation, it was hypothesized that the PT relays olfactory information to downstream brainstem motor regions that produce swimming behavior. However, the mechanisms through which the PT contributes to olfactory-induced locomotion are not identified.

The aim of the present study in sea lampreys was first to characterize secondary olfactory projections from the medOB to the PT and identify the cellular phenotype of neurons which receive these fibers since neuronal populations within the PT are heterogeneous in neurotransmitter content [9,22]. Second, we tested whether the PT responds to olfactory inputs and whether they are relayed to brainstem motor regions to produce locomotion. Our results show the presence of dense medOB projections within the PT, close to DA, glutamatergic, and GABAergic neurons. Moreover, after confirming physiologically that PT neurons are activated by olfactory inputs, we demonstrated that both the medial and the lateral olfactomotor pathways activate the same PT neurons that project to the MLR. Dopaminergic, MLR-projecting PT neurons [21] are likely to be involved. Furthermore, we show that medOB and LPal inputs induce swimming in semi-intact preparations with the whole brain intact. Interestingly, recordings of PT activity revealed that this region is robustly recruited during locomotion evoked by either medOB or LPal stimulation. Surprisingly, we also observed that the PT is systematically activated throughout every swimming episode, including spontaneously occurring locomotion. This suggests that PT activity plays an important role during locomotion. Altogether, our results show that the activation of neuronal populations in the PT upon stimulation of the medial and lateral olfactomotor pathways induces locomotion, presumably through projections to the MLR.

## 2. Results

### 2.1. The medOB Projects to DA Neurons of the PT

**Figure 1:**
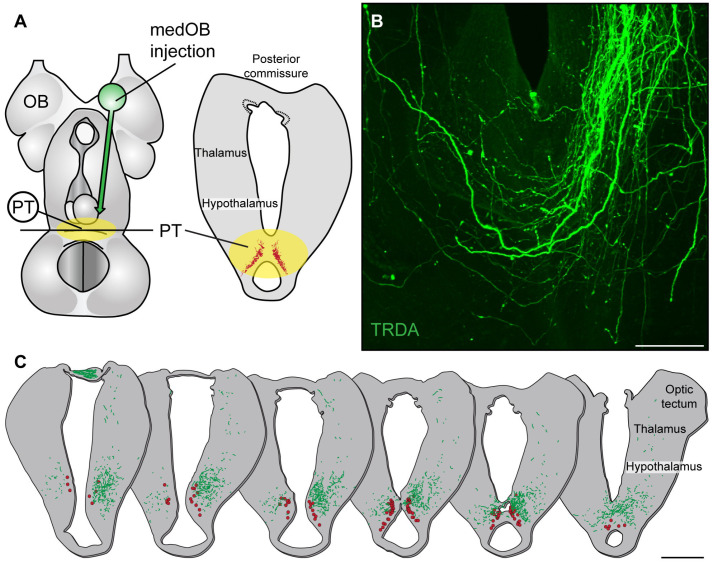
Localization of medOB projections close to DA neurons in the PT. (**A**) The schematic dorsal view of the adult lamprey forebrain and mesencephalon shows the injection site in the medial olfactory bulb (medOB) and the rostrocaudal level of the posterior tuberculum (PT) at the mesodiencephalic junction. The adjacent schematized transverse section shows the location of dopaminergic (DA) neurons in the PT (red). (**B**) Confocal image representing a horizontal section at the level of the PT (rostral is at the top) in a spawning phase adult. Photomicrographs were taken in a whole brain that was cleared with methylsalycylate following a Texas Red-conjugated dextran amine (TRDA; 3000 MW; depicted in green) injection in the medOB that labeled dense varicose projections ending in the PT. (**C**) Illustrations of serial transverse sections at the level of the PT (distance between sections: 50 μm) with superimposed DA cell bodies (enlarged red circles) and medOB projections that were anterogradely labeled with TRDA (green) in a spawning phase adult. Scale bar in B: 100 µm; scale bar in C: 500 µm.

**Figure 2:**
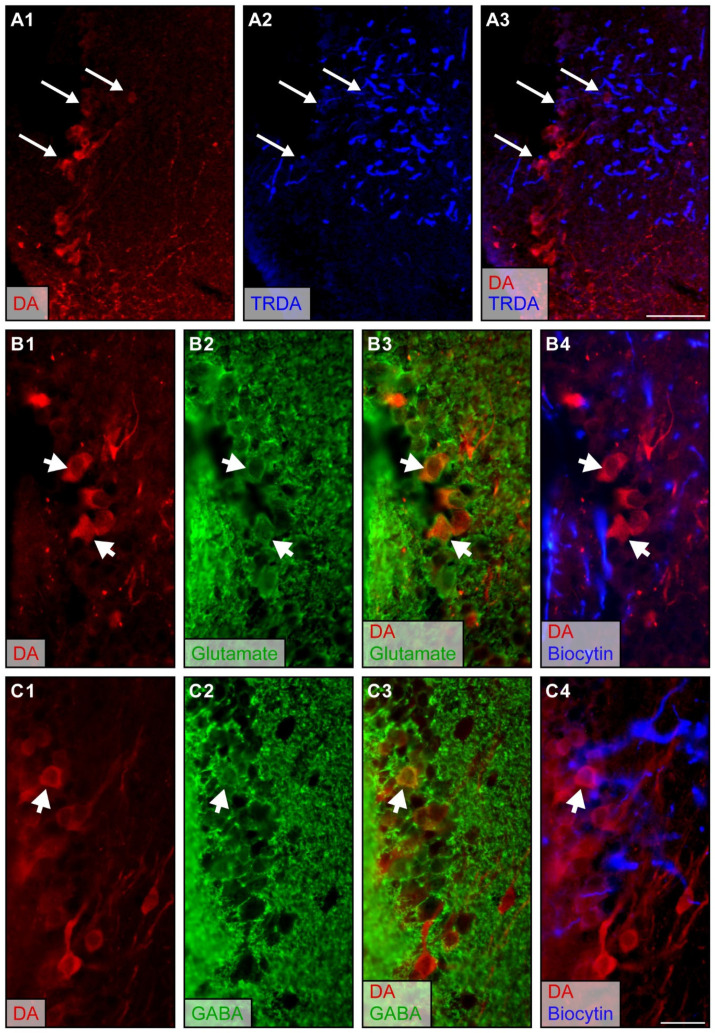
Secondary olfactory projections from the medOB in close proximity to DA, glutamatergic, and GABAergic neurons in the PT. All photomicrographs were taken in transverse sections at the level of the PT in adult lamprey brains. Biocytin injection in the medOB labeled fibers projecting to the PT that were observed in combination with immunofluorescence. (**A1**–**A3**) Confocal images representing DA immunofluorescence (depicted in red) in the PT superimposed with TRDA-labeled (depicted in blue) medOB projections showing close proximity (white arrows) in a spawning phase adult. (**B1**–**B4**) Photomicrographs showing the presence of DA-immunopositive (**B1**, red) and glutamate-immunopositive (**B2**, green) neurons in the PT, some of which are co-labeled (**B3**, white arrowheads) and in close proximity with axonal projections from the medOB (**B4**, blue, white arrowheads) in a spawning phase adult. (**C1**–**C4**) Photomicrographs showing the presence of DA-immunopositive (**C1**, red) and GABA-immunopositive (**C2**, green) neurons in the PT, some of which are co-labeled (**C3**, white arrowheads) and in close proximity with axonal projections from the medOB (**C4**, blue, white arrowheads) in a newly transformed adult. Scale bar in A3: 50 µm; scale bar in C4: 25 µm.

In the sea lamprey, anatomical experiments were first carried out to characterize medOB projections to the PT and examine the possibility that the PT transmits olfactomotor signals from the medOB to downstream motor centers. Texas Red-conjugated dextran amines (TRDA) were injected into the medOB, and anterograde labeling confirmed the presence of descending projections to the PT (Figure 1). We used whole brain preparations cleared in methyl salicylate (*n* = 2 spawning phase adults, 1 newly transformed adult) to observe in the horizontal plane the entire fiber bundle originating from the medOB and projecting to the PT (Figure 1B). This allowed us to obtain an overall representation of fibers reaching the PT. While most medOB projections were varicose and ended in the ipsilateral PT, some also crossed the midline to reach the contralateral PT and hypothalamus. To determine if medOB projections may reach DA neurons in the PT, like MOB [32] and LPal [7] projections, anterograde tracer injections in the medOB were performed in combination with immunofluorescence directed against DA (red in Figure 1C; *n* = 14 spawning phase adults, 17 newly transformed adults, 2 larvae). The tissue was examined in histological sections. Descending projections were denser on the ipsilateral side as large varicose fibers turned medially from the hypothalamus and extended bilaterally within the PT, intermingled with DA and non-DA neurons. Moreover, confocal microscopy allowed us to observe the superposition of anterogradely labeled medOB varicose projection endings and DA-immunopositive somata and neurites in z-projection images at the level of the PT (arrows in Figure 2A). These results suggest that medOB projection neurons directly transmit the olfactory signal to DA neurons on both sides of the PT.

Moreover, it was recently shown that DA neurons of the PT can co-store glutamate and/or GABA [9,22], such as in the mammalian SNc/VTA [51,52]. Thus, double immunofluorescence protocols directed against DA and glutamate (*n* = 2 spawning phase adults; 4 newly transformed adults) or DA and GABA (*n* = 8 newly transformed adults) were performed to identify the neuronal phenotype of PT cells in relation to medOB projections. Anterograde tracing revealed that medOB fibers are in close proximity to co-labeled DA/glutamate neurons (arrowheads in Figure 2B) and DA/GABA neurons (arrowheads in Figure 2C). This suggests that DA neurons co-storing glutamate and/or GABA receive olfactory inputs from the medOB.

### 2.2. Stimulation of the Olfactomotor Circuitry Induces Neuronal Responses in the PT

**Figure 3:**
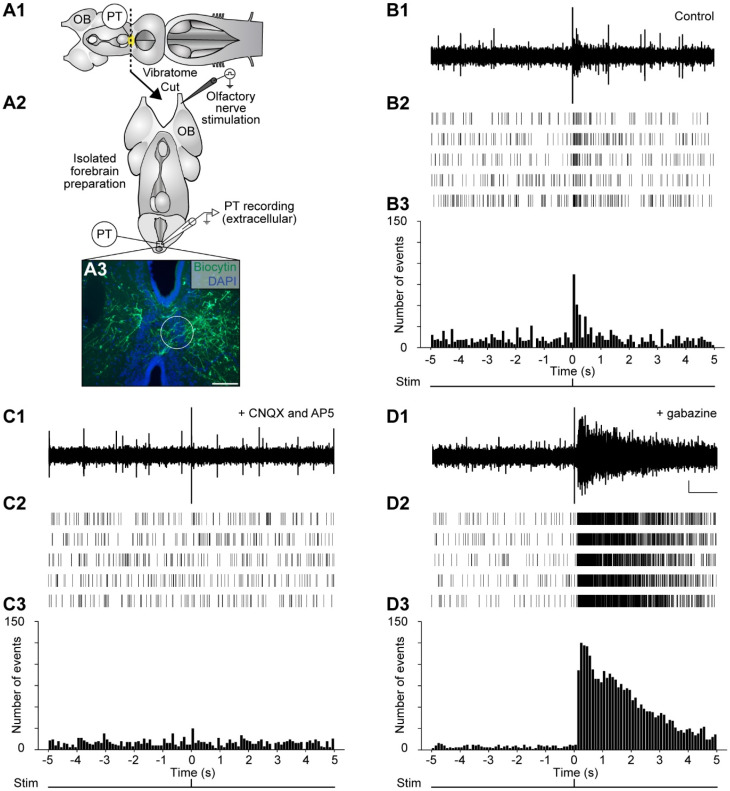
Extracellular responses in the PT to electrical stimulation of the olfactory nerve. (**A1**) The schematic dorsal view of the isolated adult lamprey brain illustrates the rostrocaudal level at which a transverse section was made to produce the isolated forebrain preparation (**A2**) that enables experimental access to the PT. (**A3**) Photomicrograph of a transverse section at the level of the PT illustrating the extracellular recording site (white circle; tip diameter: 125 µm). Cell populations within the PT are labeled with 4′,6-Diamidino-2-Phenylindole (DAPI; blue), and axonal projections of the medOB (green) are anterogradely labeled by a biocytin injection. (**B1**) Extracellular recording in the PT shows responses evoked by electrical stimulation of the ipsilateral olfactory nerve. In a representative animal, five responses are shown in a raster plot (**B2**) aligned on the time of stimulation (time = 0 s) and summed in a vertical bar chart (**B3**, bar width: 100 ms). The spikes occurring at time = 0 s are stimulation artifacts and have not been included in the histograms. (**C1**–**C3**) The same representation is shown in the same animal following local pressure ejection of a combination of glutamatergic receptor antagonists (6-cyano-7-nitroquinoxaline-2,3-dione, CNQX: 1 mM; DL-2-amino-5-phosphonopentanoic acid, AP5: 0.5 mM) in the PT, which abolishes responses induced by electrical olfactory nerve stimulation. (**D1**–**D3**) In a different animal, bath application of the GABA_A_ receptor antagonist (gabazine: 10 µM) dramatically increased extracellular responses evoked by the electrical stimulation of the olfactory nerve. Scale bar in A3: 100 µm; scale bars in D1: 50 µV and 1 s.

We then assessed whether neuronal responses are induced in the PT by olfactory nerve stimulation. Neural activity was thus recorded extracellularly in the PT of isolated forebrain preparations in response to electrical stimulation of the olfactory nerve (Figure 3A). The recording electrode (tip diameter: 125 µm) was positioned over the population of DA neurons intermingled with descending projections of the medOB that we previously identified (see Figure 1 and Figure 2). Upon electrical stimulation (single 2 ms square pulse, 10–30 µA) of the olfactory nerve, bursts of activity were evoked (Figure 3B; *n* = 11 newly transformed adults). Moreover, when glutamate receptor antagonists (competitive AMPA/kainate receptor antagonist 6-cyano-7-nitroquinoxaline-2,3-dione, CNQX: 1 mM; competitive NMDA receptor antagonist DL-2-amino-5-phosphonopentanoic acid, AP5: 0.5 mM) were locally microinjected in the PT, no response to electrical stimulation of the olfactory nerve could be observed (Figure 3C; *n* = 2 newly transformed adults). This confirms that the descending olfactory projections to the PT are glutamatergic [24,30]. In contrast, when GABA_A_ receptor antagonists (gabazine: 10 µM) were applied to the bath, a striking increase in PT activity was observed in response to olfactory nerve stimulation (Figure 3D; *n* = 7 newly transformed adults), which is consistent with a tonic GABAergic inhibition in the OB [30]. These results confirm that PT neurons are depolarized by olfactory stimulation.

**Figure 4:**
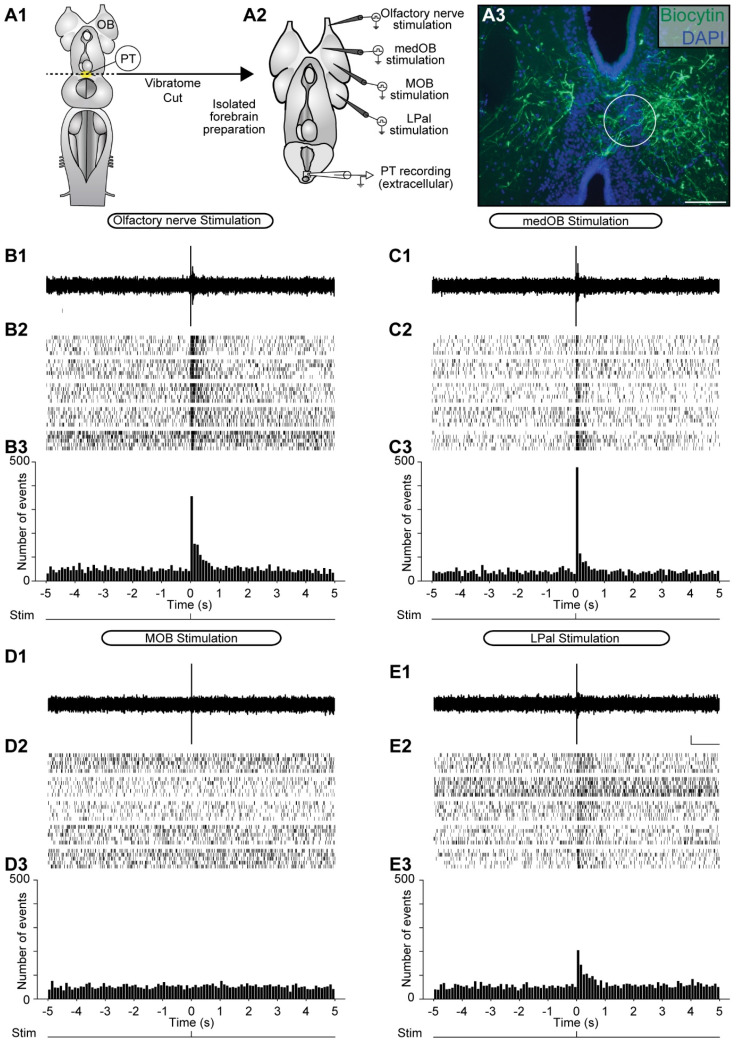
Extracellular responses in the PT to electrical stimulation of the olfactory nerve, medOB, MOB, and LPal. (**A1**) The schematic dorsal view of the isolated adult lamprey brain illustrates the rostrocaudal level at which a transverse section was made to produce the isolated forebrain preparation (**A2**) that enables extracellular recording in the PT and electrical stimulation of the olfactory nerve (**B1**–**B3**), medOB (**C1**–**C3**), main olfactory bulb (MOB; **D1**–**D3**), and lateral pallium (LPal; **E1**–**E3**). (**A3**) Photomicrograph of a transverse section at the level of the PT illustrating the extracellular recording site (white circle; tip diameter: 125 µm). Cell populations within the PT are labeled with DAPI (blue), and axonal projections of the medOB (green) are anterogradely labeled by a biocytin injection. (**B1**) Extracellular recording in the PT shows the response evoked by electrical stimulation of the ipsilateral olfactory nerve in a representative animal. (**B2**) In a raster plot, 25 responses from 5 newly transformed adults are aligned on the time of stimulation (time = 0 s) and summed in a vertical bar chart (**B3**, bar width: 100 ms). The spikes occurring at time = 0 s are stimulation artifacts and have not been included in the histograms. The same organization is shown with the same representative animals after the stimulation electrode was repositioned in the ipsilateral medOB (**C1**–**C3**), MOB (**D1**–**D3**), or LPal (**E1**–**E3**). Scale bar in A3: 100 µm; scale bars in E1: 50 µV and 1 s.

**Figure 5:**
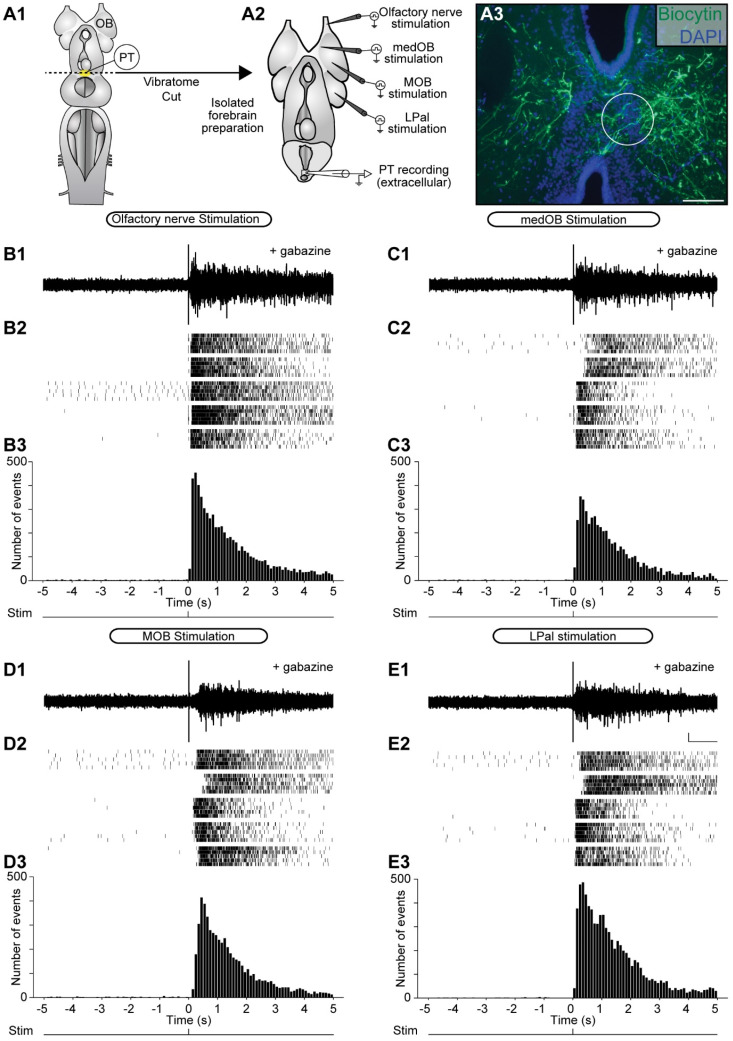
Extracellular responses in the PT to electrical stimulation of the olfactory nerve, medOB, MOB, and LPal during the bath application of gabazine. (**A1**) The schematic dorsal view of the isolated adult lamprey brain illustrates the rostrocaudal level at which a transverse section was made to produce the isolated forebrain preparation (**A2**) that enables extracellular recording in the PT and electrical stimulation of the olfactory nerve (**B1**–**B3**), medOB (**C1**–**C3**), MOB (**D1**–**D3**), and LPal (**E1**–**E3**). (**A3**) Photomicrograph of a transverse section at the level of the PT illustrating the extracellular recording site (white circle; tip diameter: 125 µm). Cell populations within the PT are labeled with DAPI (blue), and axonal projections of the medOB (green) are anterogradely labeled by a biocytin injection. (**B1**) Extracellular recording in the PT shows the amplified response evoked by electrical stimulation of the ipsilateral olfactory nerve in a representative animal during the bath application of GABA_A_ receptor antagonists (gabazine: 10 µM). (**B2**) In a raster plot, 25 responses from 5 newly transformed adults are aligned on the time of stimulation (time = 0 s) and summed in a vertical bar chart (**B3**, bar width: 100 ms). The spikes occurring at time = 0 s are stimulation artifacts and have not been included in the histograms. The same organization is shown with the same representative animals after the stimulation electrode was repositioned in the ipsilateral medOB (**C1**–**C3**), MOB (**D1**–**D3**), or LPal (**E1**–**E3**), all of which also evoke amplified extracellular responses in the PT. Scale bar in A3: 100 µm; scale bars in E1: 50 µV and 1 s.

Stimulation of the olfactory nerve recruits both the medial [24] and the lateral [30] olfactomotor pathway by activating the medOB and the MOB. To compare the PT responses to stimulation of these pathways, the extracellular electrode was kept in the same PT recording site while consecutively stimulating the olfactory nerve, medOB, MOB, and LPal (Figure 4, *n* = 5 newly transformed adults). Upon electrical stimulation of the ipsilateral medOB (Figure 4C), bursts of neural activity were observed in the PT, which confirms that medOB projections can indeed induce excitatory responses in the PT. Moreover, similar PT responses were recorded following electrical stimulation of the LPal (Figure 4E). Interestingly, similar bursts of activity were also observed in the contralateral PT (Figure S1, *n* = 3 newly transformed adults) upon stimulation of the olfactory nerve, medOB, and LPal. However, upon stimulation of the MOB, which could be anatomically coupled to PT neurons through a relay in the LPal [30], no responses were observed in the ipsilateral (Figure 4D) or contralateral PT (Figure S1D). Previous results [30] showed that MOB stimulation does not induce RS cell responses because it is under a strong GABAergic inhibitory control. Hence, we tested the effect of bath-applied gabazine (10 µM) and found that large bursts of neural activity are induced following electrical stimulation of the MOB (Figure 5D). In addition, responses to the stimulation of the olfactory nerve (Figure 5B), medOB (Figure 5C), or LPal (Figure 5E) were also increased during the bath application of gabazine. Responses to the different stimulation sites showed variability in their onset. Because this was not consistent for all the animals illustrated, we have not analyzed this further.

**Figure 6:**
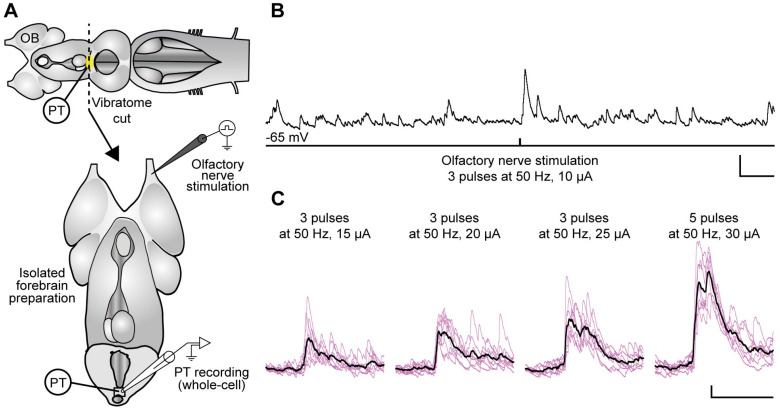
Whole-cell responses in the PT to electrical stimulation of the olfactory nerve. (**A**, **top**) The schematic dorsal view of the isolated adult lamprey brain illustrates the rostrocaudal level at which a transverse section was made to generate an isolated forebrain preparation (**A**, **bottom**) that allows access to the PT. (**B**) Whole-cell patch clamp recording (current clamp mode, zero current) of a neuron within the DA nucleus of the PT shows spontaneous synaptic activity and a response to electrical stimulation of the olfactory nerve. (**C**) In the same representative animal, excitatory postsynaptic potentials were evoked by stimulations of increased intensities. Responses are represented as eight superimposed traces (colored) and their mean (thick black trace). Scale bars in (**B**): 10 mV and 1 s; scale bars in (**C**): 2 mV and 1 s.

Next, to determine how PT neurons react to olfactory inputs, intracellular recordings were performed within the DA nucleus of the PT of the isolated forebrain preparation (Figure 6A). Whole-cell patch clamp recordings of PT neurons (*n* = 45 neurons in 13 newly transformed adults) were performed to assess the presence of synaptic responses to electrical stimulation of the olfactory nerve (trains of 1–5 pulses, 50 Hz, 7.5–20 µA). Synaptic responses were observed in 8 cells from 6 animals (Figure 6), whereas 37 cells from 12 animals did not respond to the stimulation. Synaptic responses showed variation from cell to cell as both excitatory (6 out of 8 cells) and inhibitory (2 out of 8 cells) postsynaptic potentials or currents were recorded. This variability may be explained by the heterogeneity of neuronal populations in the PT (see Figure 2) [9,22].

**Figure 7:**
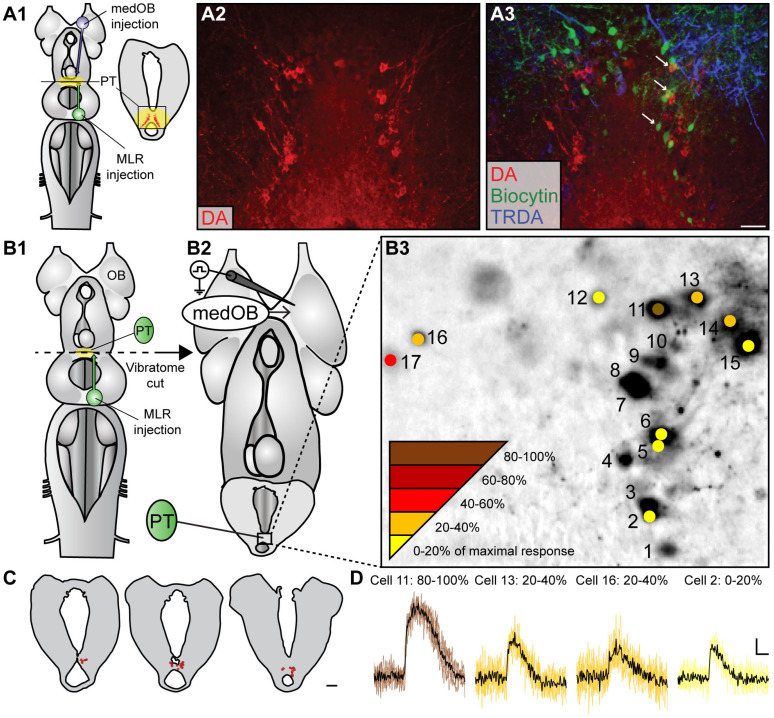
PT neurons that project to the MLR are activated by medOB input. (**A1**) The schematic dorsal view of the lamprey forebrain and mesencephalon shows the TRDA (blue) injection site in the medOB, the biocytin (green) injection site in the mesencephalic locomotor region (MLR), and the level of the PT at the mesodiencephalic junction. The adjacent schematized transverse section shows the location of the DA nucleus of the PT and the black frame corresponds to the region illustrated in (**A2**,**A3**). Immunofluorescence directed against DA (red; **A2**) labels neurons in the PT (white arrows) that are also retrogradely labeled by a biocytin injection into the MLR (green) and in close apposition to medOB projections anterogradely labeled by TRDA (depicted in blue; **A3**) in a newly transformed adult. (**B1**) The schematic dorsal view of the isolated adult lamprey brain illustrates the Calcium Green dextran crystals (green) injection in the MLR and the rostrocaudal level at which a transverse section was made to produce the isolated forebrain preparation (**B2**) that allows access to the PT. The black frame in (**B2**) corresponds to the PT region that is imaged during electrical medOB stimulation following the bath application of gabazine (5 µM). (**B3**) The image shows the mean calcium signal (as shades of gray) during a 900 s acquisition to visualize neurons (1–17) that are retrogradely labeled by the MLR injection. Out of 17 labeled cells, 10 respond to medOB stimulation, and for each of these, the area under the curve (relative changes in fluorescence, or ΔF/F, over time) was measured. The value of 100% was assigned to the cell with the maximal response (cell #11), and other cells were color coded according to their percentage of the maximal response (see the inset of (**B3**)). (**C**) Three schematized transverse sections at the level of the PT illustrate the approximate localization of MLR-projecting neurons that respond to medOB stimulation in all tested animals (30 neurons in 5 newly transformed adults). (**D**) Representative calcium responses to the same electrical medOB stimulation of four distinct cells are shown above in (**B3**). Responses are represented as six superimposed traces (colored) and their mean (thick black trace). The cell number shown above each response in panel (**D**) corresponds to the cell numbers shown in panel (**B3**). Scale bar in (**A3**): 50 µm; scale bar in (**C**): 200 µm; scale bars in (**D**): 10% ΔF/F and 10 s.

Once we confirmed that PT neurons respond to olfactory inputs, we examined whether they may subsequently recruit downstream locomotor circuitry. Therefore, we assessed whether medOB projections could recruit PT neurons that project to the MLR. In a series of anatomical experiments (Figure 7A), we injected biocytin crystals into the MLR to retrogradely label PT neurons that project to this region. Also, TRDA was injected into the medOB to label secondary olfactory projections that reach the PT, in combination with immunofluorescence directed against DA (*n* = 12 spawning phase adults, 3 newly transformed adults, 2 larvae). First, we found that many DA-immunopositive neurons were retrogradely labeled on both sides of the PT from a unilateral biocytin injection in the MLR. Similar to Ryczko and collaborators [21], retrogradely labeled DA neurons were located exclusively in the DA nucleus of the PT, mostly on the ipsilateral side, and none were found in the adjacent mammillary area, thus confirming the previous results from our laboratory. Furthermore, our experiments revealed that varicose medOB projections are close to retrogradely labeled DA-immunopositive neurons (arrows in Figure 7A), which suggests that DA neurons in the PT may relay the olfactory signal from the medOB to the MLR to induce locomotion following odor detection.

Physiological experiments were then designed to assess if PT neurons that project to the MLR respond to olfactory stimulation (Figure 7B–D; Video S1). Neurons in the PT were first retrogradely labeled by an injection of Calcium Green dextran crystals in the MLR before a transverse section was made to create an isolated forebrain preparation that enables experimental access to the PT. Also, gabazine (5 µM) was applied to the recording chamber. Unilateral electrical stimulation (1–3 pulses, 50 Hz, 5–30 µA) of the medOB induced calcium responses in neurons on both sides of the PT (*n* = 30 neurons in 5 newly transformed adults; Figure 7 and Figures S2 and S3; Video S1). Although their cellular phenotype was not ascertained, responding neurons were located within the nucleus of DA neurons of the PT (red in Figure 7C). Similarly, unilateral stimulation of the olfactory nerve (*n* = 19 neurons in 4 newly transformed adults) or the LPal (*n* = 13 neurons in 2 newly transformed adults) activated PT neurons, evoking bilateral calcium responses (Figure S3). Interestingly, out of the 13 cells that responded to LPal stimulation, 10 also responded to medOB and/or olfactory nerve stimulation (Figure S3), which suggests that individual PT neurons integrate activity from both the medial and lateral olfactomotor pathways. Hence, upon odorant detection, the medOB and LPal may recruit the same PT neurons that relay the olfactory signal to the MLR to induce locomotion.

### 2.3. Neural Activity in the PT during Locomotion Induced by the Olfactomotor Circuitry

**Figure 8:**
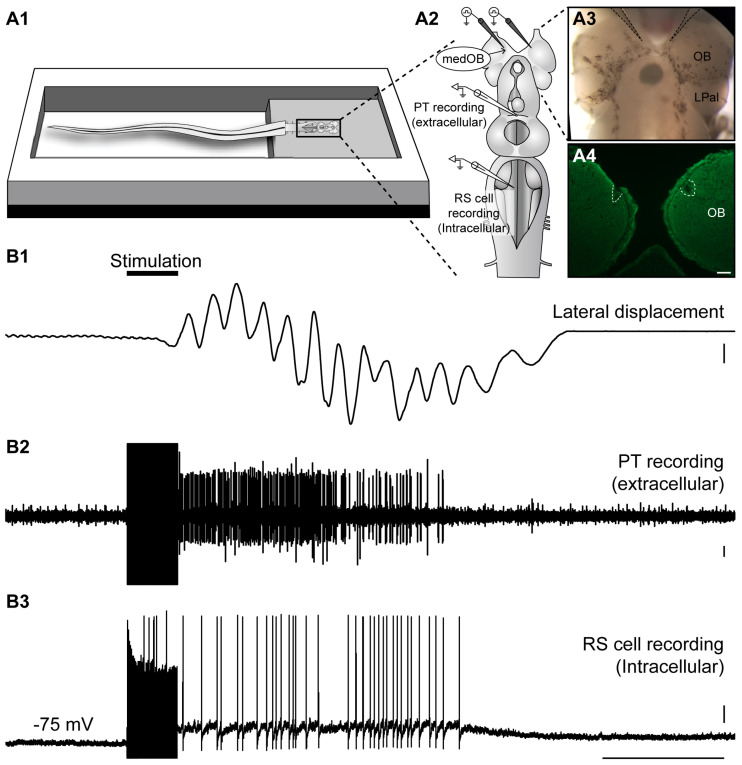
Electrical stimulation of the medOB produces swimming, extracellular activity in the PT, and spiking activity in RS cells. (**A1**) Schematized representation of the semi-intact lamprey preparation showing the isolated whole brain (black frame) pinned to the bottom of the recording chamber and the intact, freely swimming body in a second, deeper compartment; adapted from [24]. (**A2**) The brain is schematized to show the bilateral medOB stimulation site, the PT extracellular recording site, and the reticulospinal (RS) cell intracellular recording site in the middle rhombencephalic reticular nucleus (MRRN). (**A3**) Photograph of the dorsal view of the telencephalon with stimulation electrodes (dashed lines) bilaterally positioned in the medOBs. (**A4**) Photomicrograph of a transverse section at the level of the olfactory bulbs showing the lesions caused by the stimulating electrodes (white dashed lines). This confirms that the tip of both stimulation electrodes was within the medOB. (**B1**–**B3**) Bilateral medOB stimulation induced episodes of swimming activity that were accompanied by neural bursts of activity in the PT and RS cell spiking. (**B1**) Lateral displacement of a body segment was monitored with a video camera and plotted to illustrate swimming activity. Concurrently, extracellular activity was recorded in the PT (**B2**), and RS cell activity was recorded intracellularly (**B3**). Scale bar in (**A4**): 100 µm; scale bar in (**B1**): 20 mm; scale bar in (**B2**): 100 µV; scale bars in (**B3**): 10 mV and 5 s.

Next, we sought to determine whether PT neural activity may contribute to locomotion induced by olfactory stimulation. In the semi-intact preparation (intact tail freely swimming in the recording chamber), locomotion was monitored visually during intracellular recording of RS cells and extracellular recording of the PT (Figure 8A). Bilateral electrical stimulation (25 Hz, 2 s, 5–30 µA) of the medOB induced coordinated bouts of sustained locomotion that was accompanied by RS cell spiking activity and neural activity in the PT (Figure 8B and Video S2, *n* = 5 newly transformed adults). The position of both stimulation electrodes in the medOBs was histologically confirmed after the experiments (white dashed lines in Figure 8A4). Following medOB stimulation, neural activity in the PT was robustly recruited during locomotion in every animal studied, which suggests that the PT is important in inducing locomotion in the medial olfactomotor pathway.

To confirm that these responses result from medOB activity and are not generated by fibers of passage or neurons that project to the medOB, such as known PT projections to the medOB [31], the medOB was chemically stimulated bilaterally with glutamate (3–5 mM). Such stimulations induced concomitant bursts of activity in PT neurons, sustained depolarization with superimposed action potentials in RS cells, and animal swimming (Figure S4, *n* = 6 newly transformed adults). The position of glass capillaries used for glutamate injection was histologically confirmed to be in the medOBs (white dashed lines in Figure S4A). These results demonstrate that the activation of glutamatergic receptors in the medOB is sufficient to induce swimming and strongly supports our hypothesis that olfactory bulb projections recruit the PT and downstream motor centers to produce locomotion upon odorant detection.

**Figure 9:**
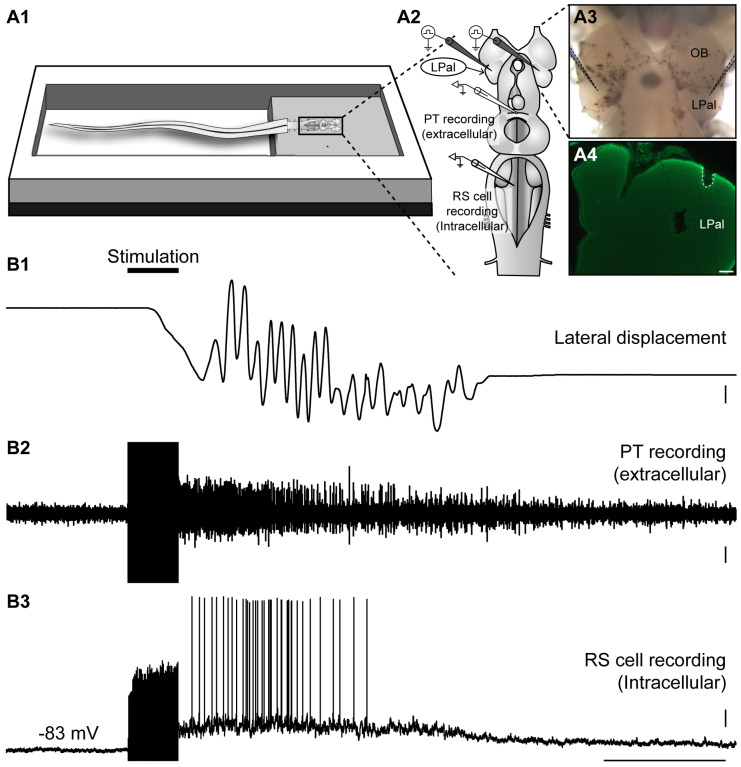
Electrical stimulation of the LPal produces swimming, extracellular activity in the PT, and spiking activity in RS cells. (**A1**) Schematized representation of the semi-intact lamprey preparation showing the isolated whole brain (black frame) pinned to the bottom of the recording chamber and the intact, freely swimming body in a second, deeper compartment; adapted from [24]. (**A2**) The brain is schematized to show the bilateral LPal stimulation site, the PT extracellular recording site, and the RS cell intracellular recording site in the MRRN. (**A3**) Photograph of the dorsal view of the telencephalon with stimulation electrodes (dashed lines) bilaterally positioned in the LPal. (**A4**) Photomicrograph of a transverse section at the level of the LPal showing the lesion caused by the stimulating electrode (white dashed line). (**B1**–**B3**) Bilateral LPal stimulation induced episodes of swimming activity with neural bursts of activity in the PT and RS cell spiking. (**B1**) Lateral displacement of a body segment was monitored with a video camera and plotted to illustrate swimming activity. Extracellular activity was concurrently recorded in the PT (**B2**), and membrane potential was intracellularly recorded in an RS cell (**B3**). Scale bar in (**A4**): 100 µm; scale bar in (**B1**): 20 mm; scale bar in (**B2**): 100 µV; scale bars in (**B3**): 10 mV and 5 s.

We then assessed whether the PT is also activated during locomotion induced by LPal stimulation. Bilateral electrical stimulation (25 Hz, 2 s, 30 µA) of the LPal reliably induced locomotion in the semi-intact preparation, simultaneously with sustained depolarization in RS cells and neural activity in PT neurons (Figure 9 and Video S3; *n* = 3 newly transformed adults). The location of the stimulation electrodes in the LPal was histologically confirmed after the experiments (white dashed line in Figure 9A4). Altogether, these results suggest that both the medOB and the LPal recruit PT neurons to induce swimming. Therefore, the PT may play an important role in locomotion evoked by both the medial and lateral olfactomotor pathways.

### 2.4. Neural Activity in the PT during Spontaneous Locomotion

**Figure 10:**
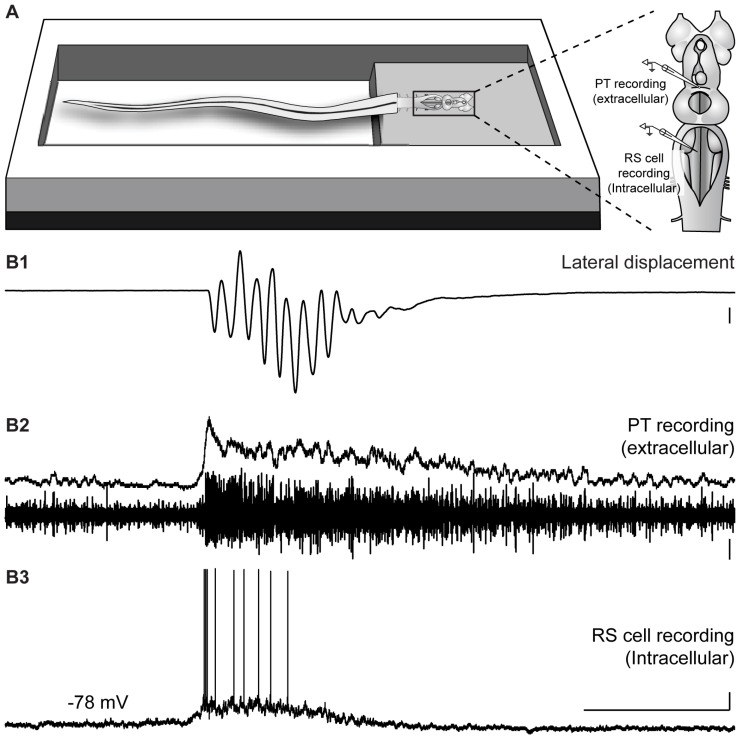
Extracellular activity in the PT and spiking activity in RS cells during spontaneous swimming in the semi-intact preparation. (**A**, **left**) Schematized representation of a semi-intact lamprey preparation showing the isolated whole brain (black frame) pinned to the bottom of the recording chamber and the intact, freely swimming body in a second, deeper compartment; adapted from [24]. (**A**, **right**) The brain is schematized to show the PT extracellular recording site and the RS cell intracellular recording site in the MRRN. (**B1**–**B3**) Episodes of spontaneous swimming were accompanied by neural bursts of activity in the PT and RS cell spiking. (**B1**) Lateral displacement of a body segment was monitored with a video camera and plotted to illustrate swimming activity. (**B2**) Extracellular recording in the PT is shown and is additionally displayed directly above as a rectified and smoothed signal. (**B3**) Intracellular recording of an RS cell in the MRRN. Scale bar in (**B1**): 20 mm; scale bar in (**B2**): 100 µV; scale bars in (**B3**): 10 mV and 5 s.

Finally, spontaneous episodes of locomotion occurred sporadically in the semi-intact preparation. Extracellular activity in the PT and intracellular activity of RS cells were recorded simultaneously during these spontaneous bouts of swimming (Figure 10, *n* = 11 newly transformed adults). Extracellular activity in the PT was invariably present during every swimming episode and was robustly coupled to RS cell and locomotor activity. The neural activity in the PT is similar in olfactory-induced (Figure 8, Figure 9 and Figure S4; Videos S2 and S3) and spontaneous (Figure 10) locomotion. This suggests the existence of a neuronal substrate at the level of the PT for locomotor control.

## 3. Discussion

**Figure 11:**
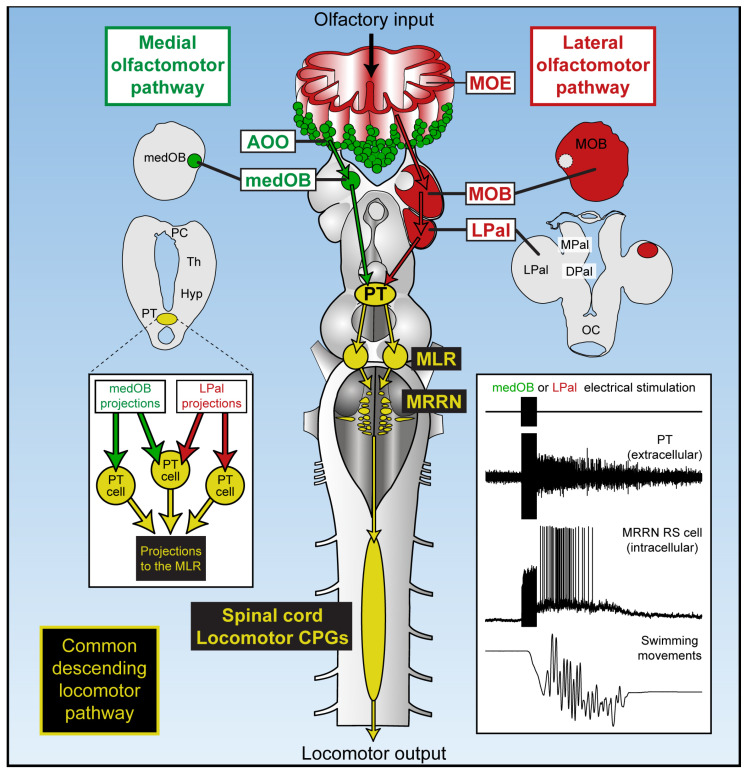
Olfactomotor circuitry in lampreys. Schematic dorsal view of the adult lamprey brain showing the hypothetic medial (green) and lateral (red) olfactomotor pathways based on [24,30,54]. Olfactory signals from the accessory olfactory organ and the main olfactory epithelium are transmitted to the medOB and MOB, respectively. The MOB then carries the olfactory signal to the LPal. We have confirmed that medOB and LPal projections activate bilateral PT neurons that reach the MLR and could, therefore, induce locomotion. These neurons may contain DA, glutamate, or GABA, or co-express DA/glutamate or DA/GABA. While some neurons projecting from the PT to the MLR respond only to inputs from the medOB or the LPal, others integrate inputs from both. This neural substrate may constitute a common descending pathway to induce locomotion in response to olfactory inputs from both the accessory olfactory organ and the main olfactory epithelium (yellow). Moreover, our data reveal that PT neurons activated by olfactory inputs project bilaterally to the MLR. We presume that following the activation by the PT, the MLR then recruits RS cells that activate the spinal locomotor central pattern generators to produce swimming movements. We have demonstrated that electrical stimulation of the medial (medOB) and the lateral (LPal) olfactomotor pathways not only induce bursts of spiking activity in RS cells and swimming of the animal but also concomitant extracellular activity in the PT that may facilitate or be necessary to produce a behavioral response following odor detection. Abbreviations: AOO, accessory olfactory organ; CPGs, central pattern generators; DPal, dorsal pallium; Hyp, hypothalamus; LPal, lateral pallium; MAM, mammillary area; medOB, medial olfactory bulb; MLR, mesencephalic locomotor region; MOB, main olfactory bulb; MOE, main olfactory epithelium; MPal, medial pallium; MRRN, middle rhombencephalic reticular formation; OC, optic chiasma; PC, posterior commissure; PT, posterior tuberculum; RS, reticulospinal; Th, thalamus.

Integrating sensory inputs into motor output is an essential function of the brain. Animals use olfaction to navigate in their environment, but the brain circuitry underlying olfactory behavior is poorly characterized. In the lamprey, two neural pathways that link the peripheral olfactory apparatus to the spinal cord motor circuitry were studied [24,29,30,31,53,54]; reviewed in [43]. Here, we delve deeper into this circuitry by describing how olfactory inputs are transmitted to the PT and transformed into motor commands. In this study, we characterized secondary olfactory projections to the PT, demonstrated that it responds to olfactory inputs, and provided evidence that this neural activity is importantly involved in producing locomotion in response to sensory inputs (Figure 11). Within the DA nucleus of the PT, we found neurons that (1) respond to olfactory inputs and (2) project to the MLR, a brainstem region controlling locomotion; reviewed in [48]. Moreover, we found individual PT neurons that are recruited by electrical stimulation of the olfactory nerve, medOB, and LPal, which may constitute a common descending pathway for the medial and lateral olfactomotor pathways to activate the MLR and downstream locomotor circuitry. Importantly, we also observed that locomotion is systematically coupled to PT activity, including spontaneously occurring swimming bouts. Therefore, a population of PT neurons may integrate external and internal inputs to induce locomotion by activating the MLR.

### 3.1. medOB Projections to DA Neurons of the PT

Direct olfactory bulb projections to the PT were anatomically documented in several lamprey species (*Ichthyomyzon unicuspis*: [27], *Lampetra fluviatilis*: [7,25,26,28,32], *Petromyzon marinus*: [24,29,30,31]), cartilaginous fishes [55], and teleostean fishes [56,57,58,59,60,61]. However, the functionality of these olfactory bulb projections to the PT was never confirmed. In the lateral olfactomotor pathway (main olfactory epithelium—MOB—LPal—PT), projections to the PT from the MOB [32] and the LPal [7,44] were previously detailed and were not investigated in our study. On the other hand, in the medial olfactomotor pathway (accessory olfactory organ—medOB—PT), the characterization of specific medOB projections to the PT was lacking until now.

One aim of the present study was to identify the cellular phenotype of PT neurons that receive medOB projections, with an emphasis on DA cells. In our material, anterograde tracing of medOB projections allowed us to observe a dense innervation of the DA nucleus of the PT (Figure 1, Figure 2 and Figure 7). Moreover, medOB tracer injection combined with confocal microscopy revealed that medOB projections ending in the PT were intermingled with DA-immunopositive neurons. The juxtaposition of medOB fibers with DA somata and dendrites means that medOB projections are positioned to directly activate them and suggests that medOB projection neurons directly relay olfactory inputs to DA neurons in the PT. Moreover, as in the PT of zebrafish [62] and mammalian SNc/VTA [51,52,63,64,65,66], DA neurons co-express other neurotransmitters, such as glutamate (Figure 2) [9,22] and GABA (Figure 2) [9]. Indeed, in the lamprey, a combination of immunofluorescence directed against tyrosine hydroxylase (the rate-limiting enzyme of DA biosynthesis) with in situ hybridization directed against the RNA of the vesicular glutamate transporter (vGluT: glutamatergic neuron marker) or the glutamic acid decarboxylase (GAD: GABAergic neuron marker) allowed von Twickel and collaborators [9] to observe different neuronal phenotypes in the PT such as DA/glutamate, DA/GABA, glutamate/GABA, and even triple-labeled DA/glutamate/GABA neurons. Since other authors could not previously observe double-labeled DA/glutamate [67] or DA/GABA [68] neurons in the PT of lampreys, it was stated that the low sensitivity of immunofluorescence may account for their negative results [9]. However, in our material, immunofluorescence was successfully used to find both DA/glutamate and DA/GABA co-labeling of neurons in the PT. Interestingly, we found that medOB projections are in close proximity to PT neurons co-expressing (1) DA/glutamate (Figure 2B) and (2) DA/GABA (Figure 2C), which suggests that these neurons respond to olfactory signals from the medial olfactomotor pathway. Based on their transmitter content and anatomical projections, these neuronal populations may process olfactory inputs differently and have distinct functions.

In the isolated forebrain preparation, we could image and precisely position pipettes into the DA nucleus of the PT, which is easily accessible for physiological recordings. This allowed us to observe calcium, intracellular, and extracellular responses of PT neurons to medOB stimulation. More importantly, in the semi-intact preparation, trains of electrical stimuli applied in the medOB induced sustained bursts of activity in the PT. Altogether, these results demonstrate anatomically and physiologically that PT neurons can indeed be activated by the medOB. However, we did not produce any definitive evidence that DA neurons are involved. That DA neurons may be activated by medOB projections is supported by the following observations: 1—anatomical projections from the medOB are in close apposition to DA neurons of the PT (Figure 1, Figure 2 and Figure 7), 2—our extracellular recording pipette was positioned over the DA nucleus of the PT (Figure 4, Figure 5 and Figure 8 and Figures S1 and S4; Video S2), and 3—calcium responses were obtained from neurons located within the DA nucleus of the PT (Figure 7 and Figures S2 and S3; Video S1). Moreover, anatomical evidence showed that MOB neurons also target DA neurons in the PT [32]. Furthermore, it is also important to note that DA neurons in the PT send projections to the medOB [31], suggesting reciprocal connections between the medOB and the PT that could allow these neurons to modulate medOB activity, and consequently, medOB inputs to the PT. Olfactory projections to the PT may have been evolutionarily conserved since, in zebrafish, all glomerular clusters send projections closely associated with DA neurons in the PT [61]. Hence, we propose that medOB projections transmit olfactory signals to DA neurons in the PT. Further studies are required to determine the exact phenotypes (glutamatergic, GABAergic, or other) of PT neurons that respond to olfactory inputs from the medOB, the MOB, and the LPal.

### 3.2. MOB and LPal Projections to the PT

Anatomical projections from the lateral olfactomotor pathway to the PT were previously described. Projections of the MOB directly reach DA neurons in the PT [32]. However, the MOB olfactory signal may reach the PT mainly through a relay in the LPal [30], which also sends efferent fibers in close apposition to DA neurons [7,44]. Since these anatomical projections were already detailed, our study did not examine them. Extracellular recordings revealed that although MOB-PT connections exist, MOB stimulation failed to elicit responses in the PT (Figure 4D and Figure S1D) except when gabazine, a GABA_A_ receptor antagonist, is added to the bath (Figure 5D). This result is consistent with the idea that a GABAergic tone exists in the olfactory bulb that acts as a “gatekeeper’’ and downregulates transmission in the lateral olfactomotor pathway [30].

In our data, stimulation of the LPal reliably induced extracellular responses in the PT, which confirms previously characterized anatomical projections in lampreys [7,44]. Similar to the medial olfactomotor pathway, LPal stimulation induces extracellular (Figure 4, Figure 5 and Figure S1) and calcium (Figure S3) responses in the PT. In the semi-intact preparation, bilateral trains of electrical stimuli applied to the LPal elicited sustained bursts of extracellular activity in the PT (Figure 9; Video S3). Altogether, our data confirm physiologically that PT neurons are activated by LPal inputs and indicate that activity in the lateral olfactomotor pathway may be processed in the PT.

In light of recent results showing that the LPal of lampreys is far more complex than originally thought and may even be considered a homolog of the mammalian neocortex (reviewed in [10]), the exact position of the stimulation electrode in the LPal during our experiments is a matter that must be addressed. Indeed, although the LPal is often considered a region with mostly olfactory functions [26,30,69,70,71,72,73,74], multiple subregions with separate afferent and efferent connectivity exist. Mainly, the LPal can be segmented into a ventral sensory area, where olfactory functions have been demonstrated [32], and a dorsal area that projects to motor centers and contains distinct visual, somatosensory, and motor areas [44,75,76]. Moreover, anatomical analysis has shown that neurons targeting the PT are located preferentially in the dorsolateral part of the LPal [44]. Interestingly, our electrical stimulation site in the LPal (shown in Figure 9A4) corresponds to this dorsolateral area where a high density of PT-projecting neurons was observed [44]. These anatomical data support our physiological results showing that PT neurons are activated by LPal stimulation.

### 3.3. Convergence of the Medial and Lateral Olfactomotor Pathways in the PT

In the lamprey, as mentioned above, two segregated olfactory subsystems were previously identified; reviewed in [43]. The medial pathway originates from the accessory olfactory organ, a distinct olfactory epithelium located in the nasal cavity that projects only to the medOB [53,54]. The lateral pathway originates from the main olfactory epithelium with projections that span all over the MOB [54]. Interestingly, inputs from the accessory olfactory organ and the main olfactory epithelium may converge in the PT, presumably to induce locomotion. Our data show that electrical stimulation of the olfactory nerve, consisting of primary afferents from both the accessory olfactory organ and the main olfactory epithelium, induces extracellular (Figure 3, Figure 4, Figure 5 and Figure S1) and intracellular (Figure 6) responses in the PT. Extracellular responses were blocked by injections of glutamate receptor antagonists in the PT (Figure 3), which is consistent with the fact that olfactory bulb projection neurons are glutamatergic [77]. The stimulation of the olfactory nerve may obviously recruit the medial and lateral olfactomotor pathways simultaneously, and the observed PT responses may correspond to the activity induced in both pathways. Indeed, when the stimulation electrode is repositioned from the olfactory nerve to the medOB or the LPal, with the same stimulation parameters, extracellular responses are also observed within the same PT recording site (Figure 4, Figure 5 and Figure S1). Moreover, in semi-intact preparations where the medOB and LPal were both stimulated, sustained extracellular responses were recorded within the same PT recording site. Furthermore, projections from the medOB (Figure 1 and Figure 2), MOB [32], and LPal [7,44] intermingle with the same population of DA neurons in the PT. Thus, the signal induced by olfactory nerve stimulation may be carried via both the medial and lateral olfactomotor pathways to the same neuronal populations inside the DA nucleus of the PT. More importantly, calcium imaging experiments demonstrated that individual PT neurons are activated by stimulation of (1) the olfactory nerve, (2) the medOB, and (3) the LPal (Figure S3). These data provide strong evidence for our hypothesis that olfactory inputs from both the accessory olfactory organ and main olfactory epithelium converge on the same DA neurons in the PT through the medial and lateral olfactomotor pathways.

Another interesting characteristic of olfactory projections to the PT is the bilateral input from the medOB and the LPal. Indeed, electrical stimulation of the contralateral olfactory nerve, medOB, or LPal also induced extracellular responses in the PT (Figure S1). Moreover, calcium imaging experiments showed that stimulation of the olfactory nerve, medOB, and LPal activate neurons in the contralateral DA nucleus of the PT (Figure 7 and Figures S2 and S3; Video S1). These data are consistent with observations that projections from the medOB (Figure 1 and Figure 7) and the LPal [44] are in close proximity to DA neurons in the contralateral PT.

### 3.4. The Role of the PT in Olfactory-Induced Locomotion

In the semi-intact preparation, electrical and chemical stimulation of the medOB and the LPal robustly induced swimming that was coupled with RS cell spiking and neural bursts of activity in the PT. Indeed, stimulation of brain regions associated with the medial or lateral olfactomotor pathways induced neural activity in the PT and RS cells that began and terminated concurrently (Figure 8 and Figure 9 and Figure S4; Videos S2 and S3). This provides strong evidence that PT activity plays a vital role in producing olfactory-induced locomotion, presumably by recruiting downstream locomotor centers. Interestingly, we have additionally demonstrated the presence of PT neurons that are recruited by olfactory inputs and also project to the MLR (Figure 7 and Figures S2 and S3), a brainstem region involved in the initiation, maintenance, and stopping of locomotion; for reviews, see [47,48,49,50]. Projections to the MLR were extensively characterized previously as PT neurons storing DA [21], glutamate, and both DA/glutamate [22] or GABA [78] were retrogradely labeled after anatomical tracer injections in the MLR. The glutamatergic projection produces a graded increase in MLR activity and swimming speed [22], while the DA projection provides additional excitation by activating D1 receptors in the MLR [21,22]. The roles of GABAergic PT neurons projecting to the MLR have not been investigated yet. Moreover, DA projections from the PT were also shown to innervate every RS cell nucleus directly, increase their activity via D1 receptors, and consequently heighten swimming speed and duration [23]. Therefore, the PT can initiate and modulate locomotor activity via DA, glutamatergic, and presumably GABAergic projections. Interestingly, we now show that projections from the medOB end in the DA nucleus of the PT, in close proximity with neurons storing DA, glutamate, GABA, DA/glutamate, and DA/GABA (Figure 1, Figure 2 and Figure 7), which makes them probable synaptic partners for the transmission of the olfactomotor signal to the MLR and RS cells. In the lateral olfactomotor pathway, it was also shown that DA neurons in the PT are in close proximity with MOB [32] and LPal [7,44] projections.

Altogether, the present results strongly support our hypothesis that DA neurons in the PT receive olfactory inputs and contribute to producing motor output by recruiting and modulating downstream motor centers, such as the MLR and RS cells. However, future experiments should test the inactivation of the PT on olfactory-induced swimming behavior to understand its role better. Moreover, chemical stimulation of the olfactory epithelium with various odorants should be tested to investigate whether specific odorants preferentially activate the PT. In the semi-intact preparation, swimming movements in response to specific odorants should also be tested. The semi-intact preparation used here with the whole brain and peripheral olfactory apparatus left attached allowed us to demonstrate for the first time that swimming is induced by stimulating the medial olfactomotor pathway and may be an essential tool for future studies of olfactory-induced behaviors. Indeed, it provides experimental access to the brain and peripheral olfactory organs while allowing analysis of body movements.

Our data reveal that the PT responds to olfactory inputs and, in turn, projects to regions involved in motor control. However, the PT is not dedicated only to processing olfactory inputs, as it receives information from several other sensory channels [8], while its efferent projection pattern enables it to exert control over many motor nuclei [7]. For instance, it was shown that the PT is activated by electrosensory stimulation applied in the surrounding bath and pulses of light delivered to the retina [8]. Interestingly, Pérez-Fernández and collaborators [7,8] also showed that the PT sends DA projections to the optic tectum that are activated by visual stimuli. Moreover, in response to visual stimuli, the optic tectum induces distinct eye and head movements that are facilitated by the DA projections from the PT. Hence, visual inputs may recruit DA neurons in the PT, which then modulate activity in the tectal circuitry to adjust visuomotor responses. We believe that, similarly, olfactory inputs to the PT recruit known descending DA projections to motor regions—such as the MLR [21,22] and RS cells [23]—to facilitate olfactomotor responses.

Furthermore, another mechanism through which the PT may affect the activity of motor nuclei is through ascending DA projections that exert control over the basal ganglia. Indeed, the basic organization of the basal ganglia was already present in lampreys and is very similar to that of mammals [5,6,7,11,12,79,80,81]; reviewed in [10,82,83]. Therefore, recruiting DA neurons in the PT may activate the lamprey homolog of the nigrostriatal pathway [1,2,12] that will act through the direct and indirect pathways to relieve the tonic inhibition maintained by basal ganglia output neurons over motor regions [84,85], such as the optic tectum and the MLR. The PT may thus participate in locomotion either via descending and/or ascending projections. First, descending projections to the MLR may be part of a common locomotor pathway that is necessary, or at least facilitating, to induce locomotion. Second, ascending DA projections to the striatum may be necessary, or at least facilitating, for disinhibition of the MLR. Because the MLR is under tonic GABAergic inhibition by the basal ganglia output nuclei [80], ascending DA projections would have to activate the direct pathway and/or deactivate the indirect pathway to disinhibit the MLR. These two hypotheses are not mutually exclusive, as both descending and ascending DA projections may be necessary to induce locomotion and because individual DA neurons in the PT have dual projections to the MLR and to the striatum [21]. In mammals, both descending DA projections to the MLR [86,87] and ascending DA projections to the striatum [88] are important for locomotor control; for reviews, see [89,90]. In the SNc, DA neurons have pro-motor functions since their activation increases the probability and vigor of movements [91], and some of them also possess dual projections to the MLR and striatum [86]. Interestingly, in Parkinson’s disease, motor deficits are associated with decreased DA innervation in the striatum [13,84,92,93] and decreased activity in the MLR [94,95,96,97]; for reviews, see [89,98]. As deficits in locomotor control occur in Parkinson’s disease [99,100,101], it is most plausible that DA neuron death induces a loss of DA input to the MLR [102], leading to decreased excitability of dopaminoceptive MLR neurons [87]. According to previously mentioned studies in lamprey [21,22], a lack of DA input to the MLR should result in a diminution of locomotor commands that contribute to locomotor deficits [87]. However, although the loss of DA innervation to the dorsal striatum has been abundantly documented [103], the extent to which the loss of DA input to the MLR contributes to the motor deficits in Parkinson’s disease remains to be established [90] and should be examined. Nonetheless, the literature reviewed here strongly suggests that meso-diencephalic DA neurons occupy important motor functions through both descending and ascending projections from basal vertebrates to mammals. In lampreys, due to the many efferent DA projections of the PT [7], much work still needs to be performed to elucidate how olfactory inputs to the PT may shape behavior.

### 3.5. The Role of the PT in Locomotion

Finally, we demonstrated that the PT is activated during spontaneously occurring swimming in the semi-intact preparation (Figure 10). Interestingly, in the same recording site within the DA nucleus of the PT, neurons are activated synchronously with locomotion that is either sensory evoked or spontaneous. This strongly suggests that the PT is systematically active during swimming and thus plays an important role in the production of locomotion. Based on our results and known PT projections [21,22,23], we propose that DA neurons are part of a common descending pathway to downstream motor centers that initiate and modulate locomotion in response to external stimuli (for example, the detection of a prey induces approach behavior) and internal stimuli (for example, hunger induces foraging). A study by Thompson and collaborators [104] supports this hypothesis. In lampreys injected with 1-methyl-4-phenyl-1,2,3,6-tetrahydropyridine (MPTP), a DA neuron-selective neurotoxin that presumably damages DA neurons in the PT, spontaneous swimming was dramatically reduced, and remarkably, the initiation and maintenance of swimming activity induced by chemical stimulation of the olfactory epithelium were also severely weakened. Since non-selective DA receptor agonist apomorphine reduced these deficits [104], DA transmission must be important for both spontaneous and olfactory-induced locomotion.

Studies in zebrafish also support our hypothesis since the DA neurons of the PT are activated by sensory stimuli, and during both spontaneous and sensory-evoked locomotion. According to detailed observations in zebrafish by Wullimann and Rink [105], the DA neurons of the PT are confined to regions identified as diencephalic clusters 2 and 4 (DC2 and DC4) [106] and may be involved in motor control, as their projections reach both the striatum [107,108] and many motor nuclei [106]. Interestingly, these neurons are activated during locomotion that occurs both spontaneously [109,110,111] and in response to sensory stimulation, such as visual [110,112,113], auditory [111], or mechanical [110] inputs. Moreover, specific photostimulation of DA neurons is sufficient to induce locomotion [111] or at least increase the probability of initiating a locomotor bout [114]. In contrast, selective ablation of DA neurons decreases locomotion that occurs spontaneously [109,111,114] and in response to sensory inputs [112,113], similar to observations made in lampreys [104]. In support of our hypothesis, this indicates an important role of the DA neurons in the PT for initiating locomotor activity that is either spontaneous or sensory evoked.

The DA neurons in the PT must play a vital role in sensorimotor integration, as they are activated by sensory inputs and act directly on motor centers, encoding both sensory and motor signals. In lampreys, neurons in the PT integrate diverse sensory information, such as olfactory (present results), visual, and electrosensory stimuli [8] and innervate many motor regions [7,8,21,22,23] that could induce or modulate motor responses following the detection of salient stimuli. The PT may, therefore, be a multisensory integration center that exerts more important sensorimotor functions than previously thought. Since the PT is also activated during spontaneous locomotion, we propose that it must be involved in adapting animal behavior to both internal and external conditions.

## 4. Materials and Methods

### 4.1. Animals

Experiments were performed on 2 larvae, 67 newly transformed adults, and 16 spawning phase adult sea lampreys (*Petromyzon marinus*, RRID:NCBITaxon_7757) of both sexes. Because differences between males and females were not expected [24,30], the sex of the animals was not taken into account. Larval animals were collected from the Pike River near Notre-Dame-de-Stanbridge, QC, Canada. Newly transformed adults were purchased from Acme Lamprey Co. (Harrison, ME, USA). Spawning phase adults were collected in the Great Chazy River (NY, USA) and kindly provided by the US Fish and Wildlife Service of Vermont. All animals were kept in aerated water maintained at 4 °C. Procedures conformed to the guidelines of the Canadian Council on Animal Care and were approved by the Université de Montréal and the Université du Québec à Montréal ethics and animal care committees. Care was taken to minimize the number of animals used and their suffering.

### 4.2. Anatomical Experiments

#### 4.2.1. Isolated Whole Brain Preparation

All animals were deeply anesthetized with tricaine methanesulfonate (MS-222, 200 mg/L, Sigma-Aldrich, St. Louis, MO, USA), and the brain was isolated in vitro in cold and oxygenated (100% O_2_) Ringer’s solution (NaCl: 130.0 mM; KCl: 2.1 mM; CaCl_2_: 2.6 mM; MgCl_2_: 1.8 mM; HEPES: 4.0 mM; dextrose: 4.0 mM; NaHCO_3_: 1.0 mM, adjusted to a pH of 7.40 with NaOH). Animals were decapitated just caudal to the heart, and all the soft tissue ventral to the notochord was removed. The spinal cord and brain were then exposed by removing the dorsal part of the vertebrae, the dorsal and lateral parts of the cranium, and the mesencephalic and rhombencephalic choroid plexuses. All cranial nerves were cautiously sectioned, except for the olfactory nerve, which was kept intact along with the peripheral olfactory organ to maintain connections between olfactory sensory neurons and the brain. Moreover, the skin of the head region was carefully removed because the skin-bound electrosensory receptive organs of the lateral line system evoked bursts of activity in PT neurons following the detection of electric fields generated by brief pulses of current applied to the surrounding bath [8].

#### 4.2.2. Tracer Injection

Crystals of biocytin (Sigma-Aldrich) or TRDA (3000 MW, Molecular Probes, Eugene, OR, USA) were used to label anterogradely medOB projections to the PT or to label retrogradely PT cell bodies with descending projections to the MLR. Tracer injection in the MLR was preceded by a midsagittal section of the rhombencephalic isthmus that enabled visualization of the injection site at the level of the I_1_ Müller cell [45]. Following careful lesion of the injection site with a fine entomological needle, crystals were immediately inserted in the tissue for 10 min. The preparation was then rinsed and transferred to a cooled chamber (8 °C) perfused with Ringer’s solution for overnight transport of the tracer.

#### 4.2.3. Dopamine, Glutamate, and GABA Immunofluorescence

Immunofluorescence directed against DA, glutamate, and GABA was performed according to procedures modified from Beauséjour and collaborators [31]. The day after the injection, brains were fixed in glutaraldehyde 2% diluted in Tris-buffered saline with low sodium metabisulfite (TBSm: 0.1% sodium metabisulfite and 0.8% NaCl diluted in Tris 0.05 M, pH 7.40) for 60 min at 4 °C, rinsed in TBSm, and incubated in sucrose (20%, diluted in TBSm) for cryoprotection. The tissue was then frozen in 2-methylbutane (−50 °C), and coronal sections (15–25 μm thickness) were produced with a cryostat, collected on ColorFrost Plus microscope slides (Thermo Fisher Scientific, Waltham, MA, USA), and dried on a warming plate at 37 °C.

Sections were rinsed (3 × 10 min) in Tris-buffered saline with high sodium metabisulfite (TBSM: 1.0% sodium metabisulfite diluted in Tris 0.05 M, pH 7.40), incubated in a reducing solution (0.2% sodium borohydride and 0.9% NaCl diluted in Tris 0.05 M, pH 7.40) for 45 min at room temperature, rinsed again in TBSM, incubated in a permeabilizing solution (10% normal goat serum and 1% Triton X-100 in TBSM) for 60 min at room temperature, and immersed in a primary antibody solution (mouse anti-DA, Millipore, 1:300, RRID:AB_94817; and/or rabbit anti-glutamate, Sigma-Aldrich, 1:600, RRID:AB_259946; and/or rabbit anti-GABA, Sigma-Aldrich, 1:300, RRID:AB_477652; diluted in the permeabilizing solution) overnight at 4 °C. The next day, sections were rinsed in TBSM with 0.1% Triton X-100 before incubation in a secondary antibody solution for 60 min at room temperature. For DA immunofluorescence, secondary antibodies (goat anti-mouse conjugated with Alexa Fluor 594, Jackson ImmunoResearch Laboratories, Inc, West Grove, PA, USA, 1:200, RRID:AB_2338881 or goat anti-mouse conjugated with Alexa Fluor 488, Invitrogen, Waltham, MA, USA, 1:200, RRID:AB_2534069) were diluted in the permeabilizing solution. For glutamate and GABA immunofluorescence, secondary antibodies (goat anti-rabbit conjugated with Alexa Fluor 488, Invitrogen, 1:200, RRID:AB_143165 or goat anti-rabbit conjugated with Alexa Fluor 350, Invitrogen, 1:200, RRID:AB_2534101) were diluted in the permeabilizing solution. Sections were then rinsed again in TBSM with 0.1% Triton X-100 and mounted with Vectashield^®^ antifade mounting medium with or without 4′,6-diamidino-2-phenylindole (DAPI; Vector Laboratories, Newark, CA, USA). Biocytin was visualized by adding streptavidin (conjugated with Alexa Fluor 488 or 350, Thermo Fisher Scientific, 1:200) to the secondary antibody solution.

#### 4.2.4. Fluorescence and Confocal Microscopy

Sections were observed on an Eclipse E600 Fluorescence Microscope (Nikon Canada, Mississauga, ON, Canada, RRID:SCR_018606) and photographed with a DXM1200 digital camera (Nikon) mounted on the microscope and driven by Automatic Camera Tamer software (Nikon, Version 2.7). Sections were also observed on a FluoView FV 1000 confocal microscope (Olympus, Shinjuku City, Japan, RRID:SCR_016840) and photographed with FluoView acquisition software (Olympus).

Photomicrographs were merged and adjusted for brightness and contrast with Photoshop CS6 software (Adobe, San Jose, CA, USA, Version 13.0.1 x64) and Fiji software (Version 1.53c) [115]. To produce schematized illustrations of immunolabeled brain sections, photomicrographs of whole brain sections were taken and assembled with the Photomerge function in Photoshop CS6, and the outline of sections and labeling were precisely drawn in Illustrator CS6 software (Adobe, Version 16.0.0 x64). The accuracy of the illustrations was validated under the microscope.

#### 4.2.5. Cleared Brain Whole Mount

To visualize medOB projections to the PT in a whole brain, a unilateral TRDA injection was performed in the medOB, followed by overnight immersion of the brain in Ringer’s solution at 8 °C, allowing axonal transport of the dye. The brain was then fixed in paraformaldehyde 4% diluted in phosphate-buffered saline (PBS: 0.1 M with 0.9% NaCl; pH 7.40) for 24 h at 4 °C and rinsed in PBS. Next, the tissue was dehydrated through successive incubations in ethanol (5 min at 50%, 5 min at 70%, 5 min at 85%, 5 min at 95%, 15 min at 100%). The dehydrated brains were then cleared and stored in methyl salicylate (Thermo Fisher Scientific). The brains were mounted ventral side up on a concave microscope glass slide for observation under a FluoView FV 1000 confocal microscope equipped with a 20× water immersion objective. Images were acquired with FluoView acquisition software (Olympus, Version 04.02.03.06), and z-projection images were produced with Fiji software.

### 4.3. Physiological Experiments

#### 4.3.1. Isolated Forebrain Preparation

The above-described dissection procedures for the isolated whole brain were used in newly transformed adults. The isolated whole brain was then glued to a homemade ramp and, in a vibratome, cut through the PT with an oblique coronal plane that leaves the habenula intact. As Ericsson and collaborators reported [116], high vibration amplitude and frequency combined with slow blade advance speed enhances cell survival and preparation durability. The isolated forebrain was then pinned down to a second homemade ramp so that the rostral side faced down and the caudal end faced up. The ramp was then pinned down in an experimental chamber perfused continuously with cooled (8–10 °C) Ringer’s solution at a rate of 4 mL/min. A minimum of 1 h of recovery time preceded experimental procedures. This preparation maintains intact connections between the peripheral olfactory organ and the PT, which enables pharmacological or electrical stimulation of the olfactory nerve, medOB, MOB, and LPal while observing the PT under the microscope for calcium imaging and electrophysiological recording.

#### 4.3.2. Electrical Stimulation

Electrical stimulation was delivered to the nervous tissue with homemade glass-coated tungsten microelectrodes (4–5 MΩ; tip exposure 40–50 µm) connected to an S88 Dual Output Square Pulse Stimulator (Grass Instruments, RRID:SCR_016192) coupled to a Model PSIU6 photoelectric stimulus isolation unit (Grass Instruments). In the isolated forebrain and isolated brain preparations, single pulses (2 ms duration, 5–30 µA intensity) or trains of 2–3 pulses (50 Hz) were applied to the olfactory nerve, medOB, MOB, and LPal. In the semi-intact preparation, trains (2 s, 25 Hz, 5–30 µA) were bilaterally applied to olfactory nerves, medOBs, MOBs, and LPals with a 20 ms delay, resulting in left–right alternating pulses. To prevent desensitization of the preparation, stimulation intensity was kept at the threshold for eliciting responses. Moreover, a minimum of 50 s of recovery time was allocated before stimulation of the isolated forebrain and isolated brain preparations, and a minimum of 10 min of recovery time was allocated before stimulation of semi-intact preparations.

#### 4.3.3. Drug Application

Drugs were pressure ejected (4 psi, 20–40 ms duration) through glass borosilicate glass micropipettes (tip diameter: 10–20 µm) positioned in the medOB or the LPal. Reproducible pressure ejections were delivered by a Picospritzer II (General Valve Corporation, Fairfield, NJ, USA), and drug solutions were colored with Fast Green FCF (Thermo Fisher Scientific), a pharmacologically inactive dye, to monitor diffusion in the tissue. For bath applications, drugs were simply added to the Ringer’s solution that was continuously perfusing the recording chamber, and a minimum of 15 min was allocated before further data collection. Drugs were stored at −20 °C and dissolved in Ringer’s solution before application. The following drugs were used: SR 95531 hydrobromide (gabazine, bath applied at 5 or 10 µM, Tocris Bioscience, Bristol, UK), 6-cyano-7-nitroquinoxaline-2,3-dione (CNQX, pressure ejected at 1 mM, Tocris Bioscience), DL-2-amino-5-phosphonopentanoic acid (AP5, pressure ejected at 0.5 mM, Sigma-Aldrich), and D-glutamate (pressure ejected at 3–5 mM, Sigma-Aldrich).

#### 4.3.4. Extracellular Recordings

Extracellular recordings of neural activity in the PT were performed with suction electrodes with borosilicate glass micropipettes (Sutter Instrument; 125 µm tip diameter) and filled with Ringer’s solution. Light negative pressure was applied to the PT to increase the signal/noise ratio. Signals (100–500 Hz bandwidth) were filtered and amplified with a Model 1800 Microelectrode AC amplifier (A-M Systems, RRID:SCR_018946) and acquired through a Digidata 1200 (Axon Instruments/Molecular Devices) coupled with Axoscope software (Axon Instruments/Molecular Devices, Version 9.2.1.8).

#### 4.3.5. Whole-Cell Patch Clamp

An isolated forebrain preparation was produced according to the above procedures and transferred under an Eclipse FN1 microscope (Nikon, RRID:SCR_014995) equipped with a 20× water immersion objective. Whole-cell patch clamp recordings of neurons in the PT were made in voltage clamp mode (−60 to −70 mV) or current clamp mode (zero current) with a Model 2400 patch clamp amplifier (A-M Systems). Patch pipettes (tip resistance: 5–8 MΩ) were pulled from borosilicate glass capillaries (outer diameter: 1.5 mm; inner diameter: 0.75 mm; World Precision Instruments, Sarasota, FL, USA) on a P-87 flaming/brown micropipette puller (Sutter Instruments, Novato, CA, USA) and filled with patch pipette solution (cesium methane sulfonate: 102.5 mM; NaCl: 1 mM; MgCl_2_: 1 mM; EGTA: 5 mM; HEPES: 5 mM; ATP: 0.3 mM; and GTP: 0.1 mM, adjusted to a pH of 7.20 with cesium hydroxide, adjusted to an osmolarity of 240 mOsm with water). Better tissue penetration by the pipette was obtained by applying light positive pressure. Brightfield microscopy enabled the visualization and targeting of neuronal cell bodies within the DA nucleus of the PT for whole-cell recordings. Signals were acquired through a Digidata 1200 coupled with Clampex 9.0 software (Axon Instruments/Molecular Devices). Data analysis was performed with Spike2 software (Cambridge Electronic Design, Milton, UK, Version 5.19, RRID:SCR_000903).

#### 4.3.6. Calcium Imaging

The whole brain of newly transformed adults was isolated as described above, and a midsagittal section of the rhombencephalic isthmus was made to enable visually guided injection of the calcium-sensitive indicator dye Calcium Green-1 dextran crystals (3000 MW, Invitrogen) in the MLR at the level of the I_1_ Müller cell [45]. The preparation was then carefully rinsed and transferred to a cooled (8 °C) chamber perfused continuously with Ringer’s solution for 3–20 h, allowing the dye to backfill PT neurons projecting to the MLR. An isolated forebrain preparation was then produced according to the above procedures and mounted under an Eclipse FN1 microscope equipped with a 20× water immersion objective and a CoolSNAP HQ CCD monochrome camera (Roper Scientific GmbH, Planegg, Germany). Images were captured (2 Hz) with Metafluor^®^ Fluorescence Ratio Imaging Software version 11.0 (Molecular Devices, RRID:SCR_014294) and analyzed with Fiji software. Briefly, stacks of images were processed first with the Image stabilization plugin [117], then the Bleach correction function, and finally the Subtract background function. The mean fluorescence signal intensity of regions of interest (precisely hand drawn over dye-filled PT neuron somata) was then measured with the ROI Manager tool. Relative changes in fluorescence (ΔF/F) were calculated with the baseline (F), which is defined as the averaged fluorescence value for 50 s before stimulation.

#### 4.3.7. Intracellular Recordings (Sharp Glass Microelectrodes)

Reticulospinal cells were impaled with sharp borosilicate glass pipettes (80–120 MΩ) pulled from borosilicate glass capillaries (outer diameter: 1.5 mm; inner diameter: 0.75 mm; World Precision Instruments) on a P-87 flaming/brown micropipette puller and filled with potassium acetate (4 M). Signals were amplified with an Axoclamp 2A amplifier (Axon Instruments/Molecular Devices) and acquired through a Digidata 1200 coupled with Axoscope software. Reticulospinal cells included in this study had a stable resting membrane potential under −75 mV throughout the recording. For reproducibility of the results, only the largest Müller cells of the middle rhombencephalic reticular nucleus (MRRN; B1, B3, and B4) were recorded. Data analysis was performed with Spike2 software and a homemade script for excitatory postsynaptic potentials (created by Jean-François Gariépy).

#### 4.3.8. Semi-Intact Preparation

Semi-intact preparations were used to record neural activity in the PT and RS cells during olfactory-induced locomotion. The brain was dissected with the above-described procedures for the isolated whole brain preparation, but the body caudal to the heart was left intact and free to swim in a second, deeper compartment of a video-monitored recording chamber. A minimum of 1 h of recovery time preceded experimental procedures.

#### 4.3.9. Kinematic Analysis

Movements produced by the semi-intact preparations were recorded (30 frames per second) with an HDR-XR200 digital camcorder (Sony, Toronto, ON, Canada) positioned above the preparation. The analysis of videos was performed with a two-dimensional motion tracking software (Tracker, Open Source Physics, Version 5.1.3). Briefly, we measured the lateral displacement of a body segment along a line perpendicular to the longitudinal axis of the animal. This lateral displacement was plotted over time and displayed graphically to represent body movement. Swimming was defined as traveling mechanical waves of lateral displacement propagating from head to tail [45]. The locomotion of fish may be considered a typical undulating movement, its main feature being the waves of contractions propagating along the segments of the body musculatures [118].

## 5. Conclusions

In the lamprey, two distinct sensory epithelia exist in the peripheral olfactory organ [53,119,120] and give rise to separate olfactory pathways [53,54] that both have projections to the PT, the homolog of the mammalian SNc/VTA, which was proposed to produce locomotor output in response to olfactory inputs [24,30]. The present study shows anatomically and physiologically how the PT integrates information from both the medial and lateral olfactomotor pathways. Neurons in the DA nucleus of the PT receive bilateral input from both the medOB and the LPal and project down to the MLR, a brainstem region well known to induce locomotion. Glutamatergic, GABAergic, and mostly DA neurons may be involved. However, further experiments must be performed to confirm this. Moreover, we now demonstrate that electrical and chemical stimulation of the medOB elicits swimming in a semi-intact preparation. Interestingly, neural activity in the PT is tightly coupled to RS cell activity and undulatory swimming movements of the preparation during locomotion induced by olfactory inputs or occurring spontaneously. The PT may thus provide a common neural pathway for inducing locomotion. The results presented here provide additional insight into the neural circuits that produce motor responses following odorant detection.

## Data Availability

The data presented in this study are available upon request from the corresponding author. The data are not publicly available due to the data in this study providing data support for future laboratory experiments.

## Supplementary Materials

The following supporting information can be downloaded at https://www.mdpi.com/article/10.3390/ijms25179370/s1.
