# Supplementary material for: Olfactory Projections to Locomotor Control Centers in the Sea Lamprey
Source: Int J Mol Sci. 2024 Aug 29;25(17):9370. doi: 10.3390/ijms25179370 (PMC11395479; doi:10.3390/ijms25179370)
Supplement: Supplementary file 1 [file ijms-25-09370-s001.zip › ijms-3127210-supplementary.pdf]

Olfactory Projections to Locomotor Control Centers in the Sea Lamprey

Philippe-Antoine Beauséjour [1], Jean-Christophe Veilleux [2], Steven Condamine [1], Barbara S. Zielinski [3] and Réjean Dubuc [1,2,*]

[1] Department of Neurosciences, Faculty of Medicine, University of Montreal, Montreal, QC H3T 1J4, Canada

[2] Research Group in Adapted Physical Activity, Department of Exercise Sciences, Faculty of Sciences, University of Quebec in Montreal, Montreal, QC H2X 1Y4, Canada

[3] Department of Integrative Biology, Faculty of Science, University of Windsor, Windsor, ON N9B 3P4, Canada

* Correspondence: rejean.dubuc@gmail.com

Supplementary Figures

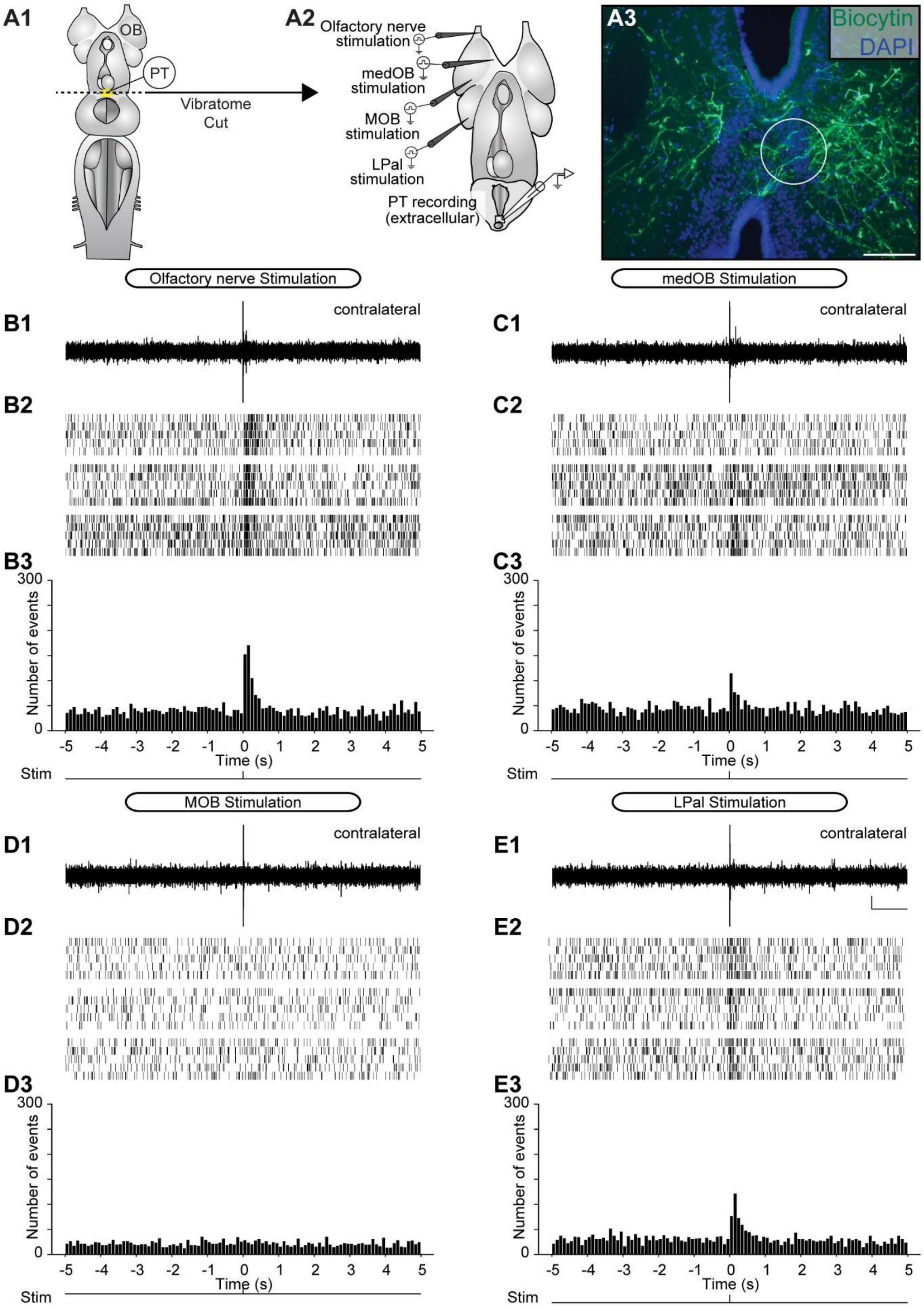

Figure S1. Extracellular responses in the PT to electrical stimulation of the contralateral olfactory nerve, medOB, MOB, and LPal. (**A1**) The schematic dorsal view of the isolated adult lamprey brain illustrates the rostrocaudal level at which a transverse section was made to produce the isolated forebrain preparation (**A2**) that enables experimental access to the PT. The schematic dorsal view of the isolated forebrain preparation illustrates the recording site in the PT and the multiple stimulation sites in the contralateral olfactory nerve (**B1–B3**), contralateral medOB (**C1–C3**), contralateral MOB (**D1–D3**), and contralateral LPal (**E1–E3**). (**A3**) Photomicrograph of a transverse section at the level of the PT illustrating the extracellular recording site (white circle; tip diameter: 125 μm). Cell populations within the PT are labeled with DAPI (blue), and axonal projections of the medOB (green) are anterogradely labeled by a biocytin injection. (**B1**) Extracellular recording in the PT shows the response evoked by electrical stimulation of the contralateral olfactory nerve in a representative

animal. (**B2**) In a raster plot, 15 responses from 3 newly transformed adults are aligned on the time of stimulation (time = 0 s) and summed in a vertical bar chart (**B3**, bar width: 100 ms). The spikes occurring at time = 0 s are stimulation artifacts and have not been included in the histograms. The same organization is shown with the same representative animals after the stimulation electrode was repositioned in the contralateral medOB (**C1–C3**), MOB (**D1–D3**), or LPal (**E1–E3**). Scale bar in A3: 100 μm; scale bars in E1: 50 μV and 1 s.

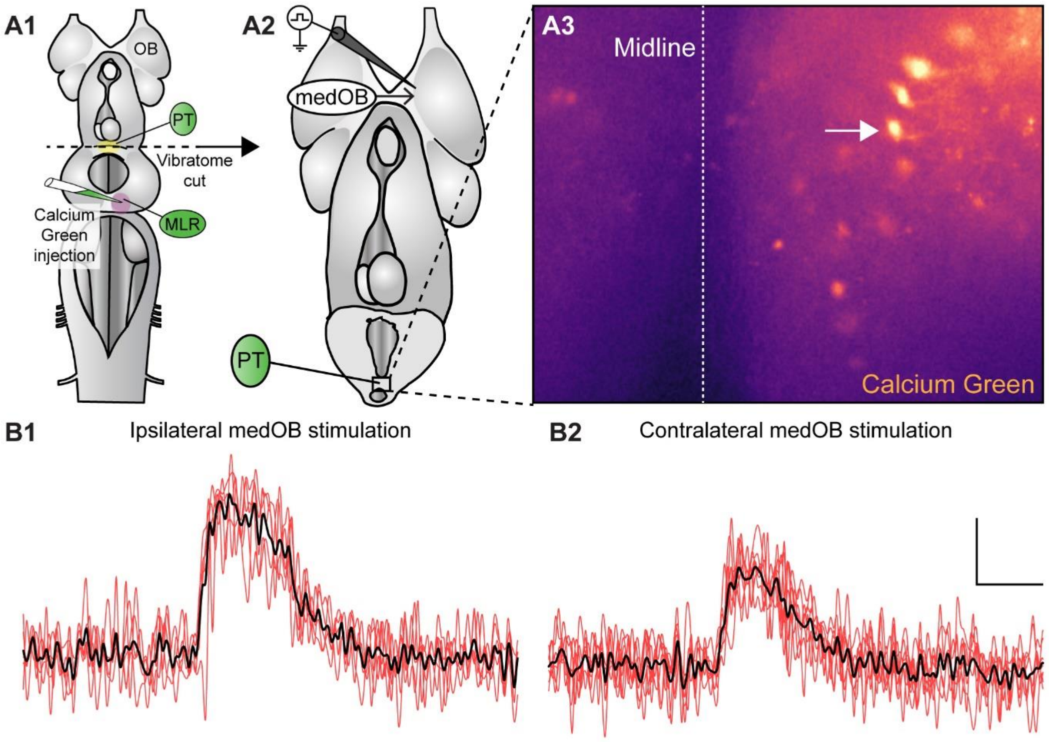

Figure S2. Stimulation of the medOB induces bilateral calcium responses in the PT. (**A1**) Schematic dorsal view of the adult lamprey brain illustrating the Calcium Green dextran amine crystals injection site in the MLR. Also shown is the rostrocaudal level of the section made to produce the isolated forebrain preparation (**A2**), which enables calcium imaging in the PT and electrical stimulation of the medOB. (**A3**) The image shows mean calcium signal, converted to shades of violet (low signal) and yellow (high signal), during a 75 s acquisition to visualize neurons that were retrogradely labeled by the Calcium Green injection in the MLR. Both traces in B1 and B2 were acquired in a single identified neuron (white arrow). (**B1–B2**) Calcium responses evoked by electrical stimulation of the ipsilateral (**B1**) and contralateral (**B2**) medOB following bath application of gabazine (5 μM) are represented as five superimposed traces (red) and their mean (thick black trace). Scale bars in B2: 10% ΔF/F and 10 s.

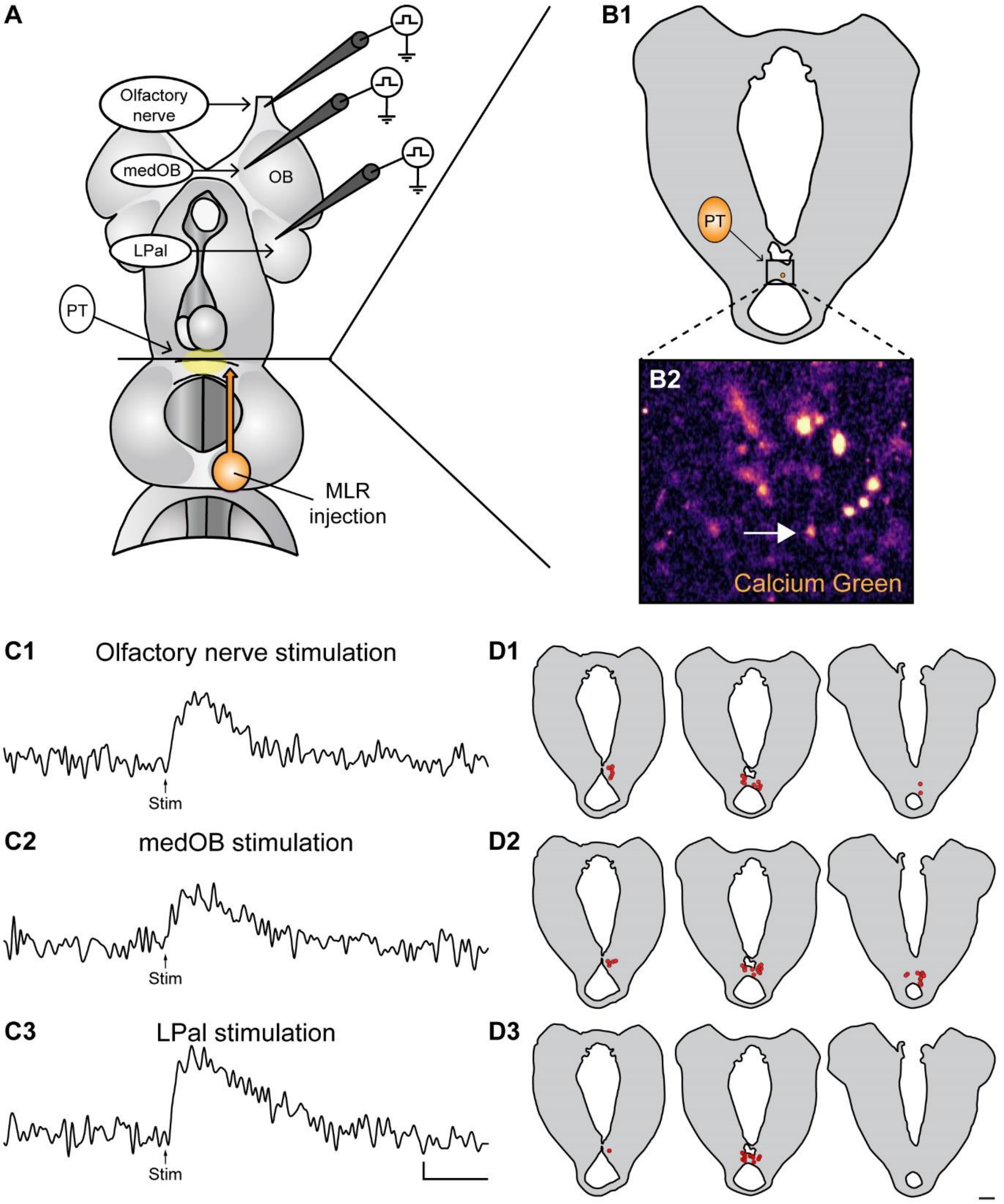

Figure S3. Stimulation of the olfactory nerve, medOB and LPal induces calcium responses in the same PT neurons. (**A**) Schematic dorsal view of the adult lamprey brain illustrating the Calcium Green dextran amine crystals injection site in the MLR and the rostrocaudal level of the section made to produce the isolated forebrain preparation. Moreover, the electrical stimulation sites in the olfactory nerve, medOB, and LPal are depicted. (**B1**) Schematized transverse section at the level of the PT, the black frame illustrates the calcium imaging site and the orange dot shows the approximate position of the imaged neuron shown in B2 and C. (**B2**) The image shows mean calcium signal, converted to shades of violet (low signal) and yellow (high signal), during a 75 s acquisition to visualize neurons that were retrogradely labeled by the Calcium Green injection in the MLR. The white arrow identifies the neuron from which the calcium responses in C were imaged. Following bath application of gabazine (5 μM), calcium responses to electrical stimulation of the ipsilateral olfactory nerve (**C1**), medOB (**C2**), and LPal (**C3**) were observed in an individual PT neuron that projects to the MLR. Traces in C1–C3 represent the mean of six calcium responses. (**D1–D3**) Schematized transverse sections at the level of the PT that represent the approximate localization of neurons (enlarged red dots) responding to the electrical stimulation of the olfactory nerve (**D1**, *n* = 19 neurons in 4 newly transformed adults), medOB (**D2**, *n* = 30 neurons in 5 newly transformed adults), and LPal (**D3**, *n* = 13 neurons in 2 newly transformed adults). Scale bars in C3: 10% ΔF/F and 10 s; scale bar in D3: 200 μm.

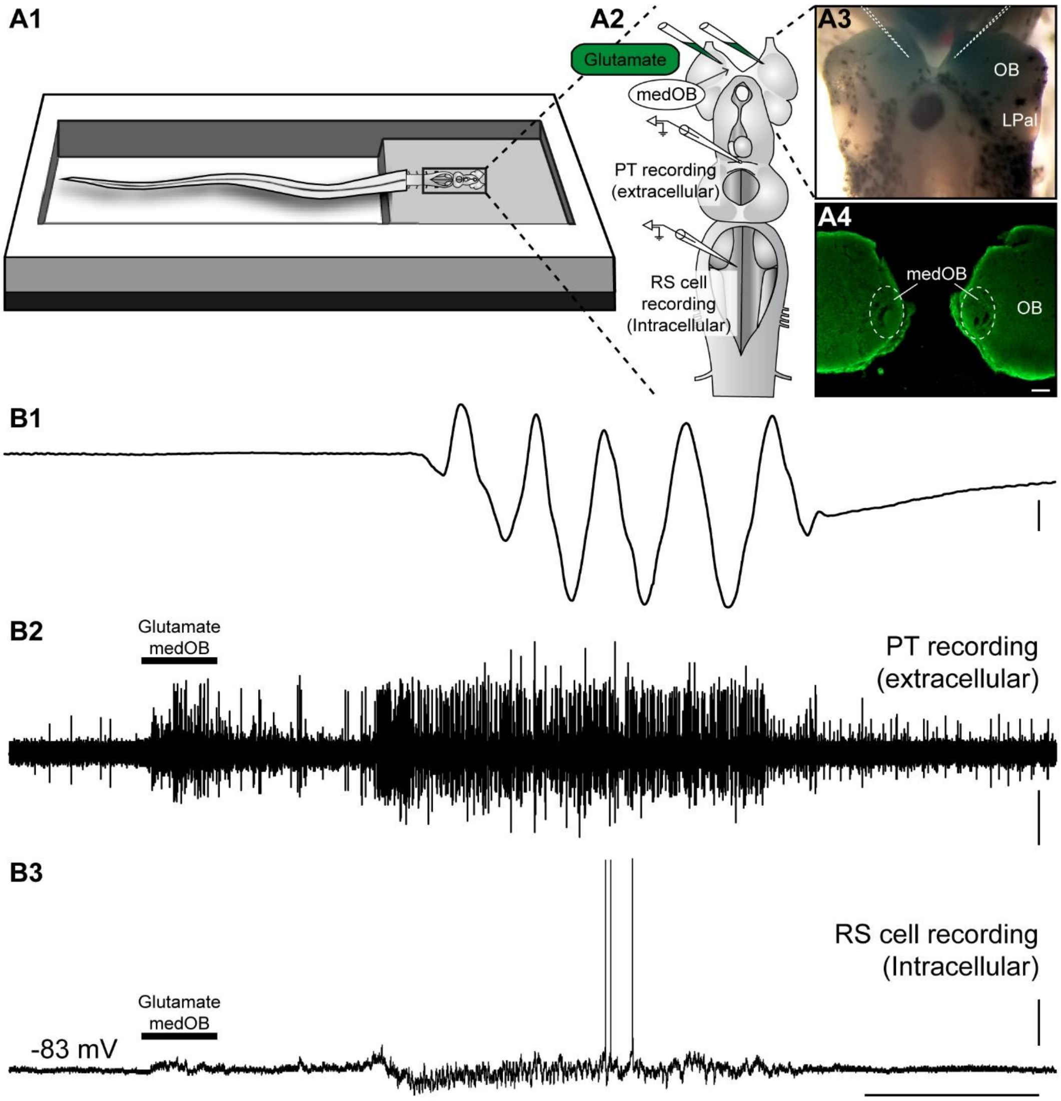

Figure S4. Chemical stimulation of the medOB produces swimming, extracellular activity in the PT and spiking activity in RS cells. (**A1**) Schematized representation of a semi-intact lamprey preparation showing the isolated whole brain (black frame) pinned to the bottom of the recording chamber, and the intact, freely swimming body, in a second, deeper compartment; adapted from [24]. (**A2**) The brain is schematized to show the bilateral glutamate injection in the medOB, the extracellular recording in the PT, and the RS cell intracellular recording in the MRRN. (**A3**) Photograph of the dorsal view of the telencephalon with microinjection pipettes (white dashed lines) bilaterally positioned in the medOBs. (**A4**) Photomicrograph of a transverse section at the level of the olfactory bulbs showing the lesions (white dashed lines) produced by the insertion of the microinjection pipettes in the medOB. This confirms that the tip of both microinjection pipettes was within the medOB. (**B1–B3**) Bilateral glutamate (3 mM) injection in the medOB induced episodes of swimming activity that was accompanied by neural bursts of activity in the PT and RS cell spiking. (**B1**) The lateral displacement of a body segment was monitored with a video camera and plotted to illustrate swimming activity. Concurrently, extracellular activity was recorded in the PT (**B2**) and RS cell activity was intracellularly recorded (**B3**). Scale bar in A4: 100 μm; scale bar in B1: 10 mm; scale bar in B2: 200 μV; scale bars in B3: 20 mV and 5 s.

Video S1. Calcium responses to medOB stimulation by neurons of the PT that project to the MLR, related to Figure 7. (**Top**) Following Calcium Green dextran amine crystals injection in the MLR, the brain was sectioned at the mesodiencephalic junction to produce an isolated forebrain preparation, which enables calcium imaging in the PT. The video in the top left panel represents the ΔF/F values measured from the calcium signal imaged in the PT during spontaneous activity and upon medOB electrical stimulation. White dashed lines over the video delineate the 3rd ventricle and the white arrow identifies the cell whose activity is shown below. (**Bottom**) The trace illustrates the calcium responses of PT neurons to medOB stimulations after two episodes of spontaneous activity. The imaged cell corresponds to cell 11 in Figure 7. The video shows an acquisition bout that lasted 450 s (imaged at 2 Hz) and compressed in 30 s (at 30 fps, played at 15× normal speed). Scale bars: 20% ΔF/F and 30 s.

Video S2. Swimming activity induced by electrical stimulation of the medOB, related to Figure 8. (**Top**) Video recording of a semi-intact preparation in which the isolated whole brain is pinned to the bottom of the recording chamber, and the intact body is freely swimming in a second, deeper compartment. Bilateral electrical stimulation (25 Hz, 2 s, 5–30 μA) of the medOB induces swimming activity (**top trace**), concurrently with bursts of neural activity in the PT (**middle trace**) and spiking activity in intracellularly recorded RS cells of the MRRN (**bottom trace**). Scale bar in the top panel: 10 mm; scale bar in the top trace: 20 mm; scale bar in the middle trace: 100 μV; scale bars in the bottom trace: 10 mV and 5 s.

Video S3. Swimming activity induced by electrical stimulation of the LPal, related to Figure 9. (**Top**) Video recording of a semi-intact preparation in which the isolated whole brain is pinned to the bottom of the recording chamber, and the intact body is freely swimming in a second, deeper compartment. (B) Bilateral electrical stimulation (25 Hz, 2 s, 5–30 μA) of the LPal induces swimming activity (**top trace**), concurrently with bursts of neural activity in the PT (**middle trace**) and spiking activity in intracellularly recorded RS cells of the MRRN (**bottom trace**). Scale bar in top trace: 5 mm; scale bar in middle trace: 100 μV; scale bars in bottom trace: 10 mV and 5 s.
